# Live Zika virus chimeric vaccine candidate based on a yellow fever 17-D attenuated backbone

**DOI:** 10.1038/s41426-018-0161-7

**Published:** 2018-09-26

**Authors:** Franck Touret, Magali Gilles, Raphaelle Klitting, Fabien Aubry, Xavier de Lamballerie, Antoine Nougairède

**Affiliations:** Unité des Virus Émergents (UVE: Aix-Marseille Univ–IRD 190–Inserm 1207–IHU Méditerranée Infection), Marseille, France

## Abstract

Zika virus (ZIKV) has recently become dispersed throughout the tropics and sub-tropics, causing epidemics associated with congenital disease and neurological complications. There is currently no commercial vaccine for ZIKV. In this study, we describe the initial development of a chimeric virus containing the prM/E proteins of a ZIKV epidemic strain incorporated into a yellow fever 17-D attenuated backbone. Using the versatile and rapid ISA (Infectious Subgenomic Amplicons) reverse genetics method, we compared different constructs and confirmed the need to modify the cleavage site between the pre-peptide and prM protein. Genotypic characterization of the chimeras indicated that the emergence of compensatory mutations in the E protein was required to restore viral replicative fitness. Using an immunocompromised mouse model, we demonstrated that mice infected with the chimeric virus produced levels of neutralizing antibodies that were close to those observed following infection with ZIKV. Furthermore, pre-immunized mice were protected against viscerotropic and neuroinvasive disease following challenge with a heterologous ZIKV strain. These data provide a sound basis for the future development of this ZIKV vaccine candidate.

## Introduction

Zika virus (ZIKV; family Flaviviridae, genus *Flavivirus*) is a single-stranded positive-sense enveloped RNA virus. The 10.8 kb ZIKV genome encodes a single polyprotein that is processed into three structural proteins (C, PrM, and E) and seven nonstructural proteins (NS1, NS2A, NS2B, NS3, NS4A, NS4B, and NS5) by viral and host proteases^1^. Phylogenetic studies have shown that all ZIKV strains characterized to date belong to two distinct lineages (African and Asian) based on the initial geographic distribution of this virus^2^. ZIKV is a mosquito-borne flavivirus transmitted primarily by *Aedes* spp. mosquitoes^3^.

Long considered to cause mild disease in humans, this arbovirus remained relatively unstudied until 2007, when it provoked a large outbreak in Micronesia^4^. Subsequently, several outbreaks occurred in different Pacific Ocean islands, including French Polynesia in 2013, where it was associated with an increased incidence of Guillain–Barré syndrome^5^. ZIKV then spread to the American continent, causing major outbreaks in Central/South America and the Caribbean and was linked with an increase of congenital neurological complications. Sexual transmission of ZIKV was also reported^6^. There is currently no commercial antiviral drug or vaccine for this virus^7^.

Several approaches are now available with which to develop inactivated^8^ and recombinant (DNA-^9^ or RNA-based^10^) ZIKV vaccines. However, live-attenuated vaccines have several advantages, including reduced costs and single-dose induction of long-term immunity^11^. Several groups developed live ZIKV vaccine candidates by making deletions in the 3′ untranslated region of the viral genome^12,13^. More recently, a chimeric ZIKV vaccine candidate based on the Japanese encephalitis virus live-attenuated strain SA14-14-2 was reported^14^. The chimeric approach had been used since the late 1990s to develop vaccine candidates against several health-threatening flaviviruses, including West-Nile virus, Japanese encephalitis virus, and all serotypes of the dengue virus^15–17^. This approach consists of incorporating prM/E of a pathogenic flavivirus in a backbone of a licensed live-attenuated vaccine strain. Indeed, E protein is prominently exposed at the surface of viral particles and is the de facto the major determinant of viral antigenicity^1^. In almost all cases, the well-characterized live-attenuated 17-D strain used to prevent yellow fever virus (YFV) infections has been used as the genetic backbone. Some of these live-attenuated vaccines are currently commercially available^17,18^.

In this study, we describe the development of a chimeric virus harboring the prM/E of an epidemic ZIKV (H/PF/2013) strain and the 17-D vaccine strain as the genetic backbone. The user-friendly and rapid ISA (Infectious Subgenomic Amplicons) reverse genetics method was used to generate the chimeric virus^19^. Finally, in cellulo and in vivo characterization of this strain demonstrated its potential as a live-attenuated vaccine candidate.

## Results

### Design and rescue of chimeric viruses

**Fig. 1:**
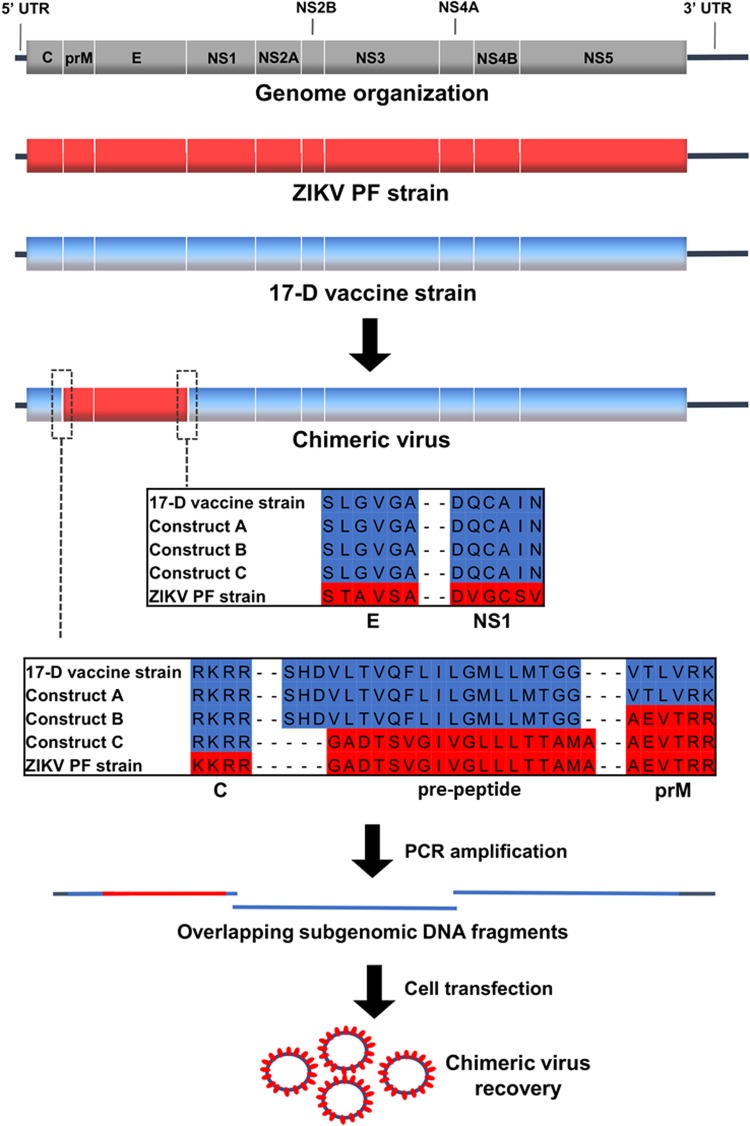
Schematic representation of the design and recovery strategies used to generate chimeric viruses. We recovered infectious virus only with construct C. The two cleavage sites are enlarged in boxes, with the amino acid alignment shown with separations between different proteins

The chimeric viruses were constructed using the yellow fever 17-D vaccine strain as a genetic backbone and by replacing prM/E from this vaccine strain with those of the Asian ZIKV PF epidemic strains. Three different constructs, designated A, B, and C, were constructed using variable sites flanking the ZIKV prM/E coding sequences (Fig. 1). Construct A harbored the pre-peptide and cleavage site before prM from the 17-D vaccine strain. Construct B harbored the pre-peptide from the 17-D vaccine strain and had a cleavage site before prM from ZIKV. Construct C harbored the pre-peptide and had a cleavage site before the prM of ZIKV. All the constructs contained the cleavage site from the 17-D vaccine strain between the E and NS1 proteins.

The ISA procedure was used to rescue the viruses. Three overlapping amplicons, which encompassed the complete genome flanked at its 5′ and 3′ extremities by the human cytomegalovirus promoter (pCMV) and the hepatitis delta ribozyme followed by the simian virus 40 polyadenylation signal (HDR/SV40pA) respectively, were transfected into a mix of HEK-293/BHK-21 cells. Because the first amplicon contained the entire structural coding region, it was only necessary to exchange the first amplicon in our previously designed and functional yellow fever 17-D vaccine strain reverse genetic system to attempt replicative virus production.

**Fig. 2:**
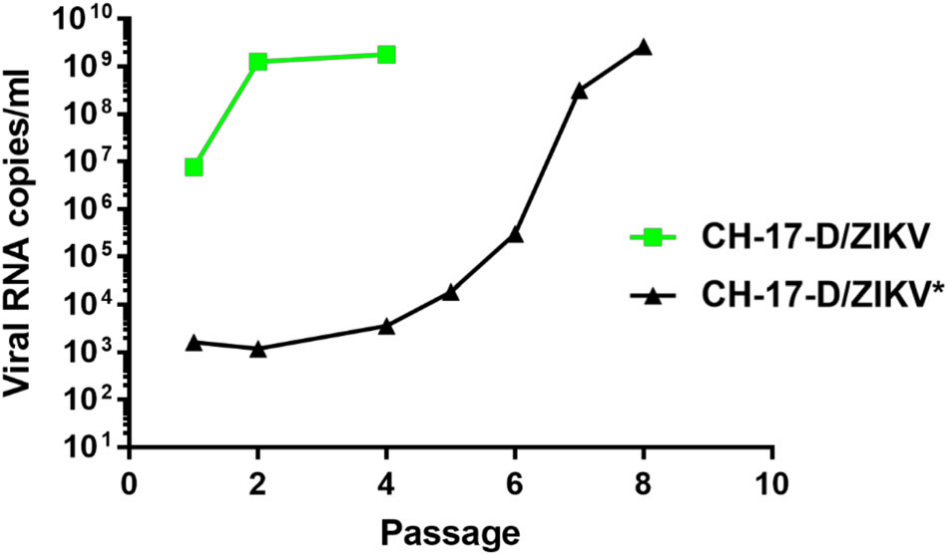
Evolution of viral production of chimeric viruses during serial passage that followed cell transfection. A mix of BHK-21/HEK-293 cells was transfected. Cell supernatant medium was subsequently passaged 4–8 times in Vero-E6 cells. Viral production in cell supernatant medium was assessed using a real-time quantitative RT-PCR assay

For each construct, we performed two independent cell transfection experiments with five replicates. After incubating for 6 days, the cell supernatant medium was passaged four times in Vero-E6 cells. Virus replication was assessed in cell supernatant medium from the last passage (passage #4) using a real-time quantitative reverse transcription PCR (qRT-PCR), and no viral replication was detected for constructs A and B. In contrast, for construct C, we detected virus replication in one well (1/5) in both independent transfection experiments. These results highlighted that the choice of the nature of the pre-peptide and cleavage site between the capsid and prM proteins is a crucial parameter when designing chimeric flaviviruses. During the first cell transfection experiment, we obtained high amounts of viral genome copies at passage #4 (1.78 e+9 viral RNA copies/ml). This virus was designated CH-17-D/ZIKV and used for in cellulo and in vivo characterizations. Surprisingly, during the second experiment, we detected very low quantities of the viral genome at passage #4 (3.57 e+3 viral RNA copies/ml), and this virus was designated as CH-17-D/ZIKV*. We next performed four additional passages using the same procedure. The quantities of viral genomes in cell supernatant medium was assessed from passage #1 to #8 and compared with that of CH-17-D/ZIKV (Fig. 2). We observed that the amount of viral genome for CH-17-D/ZIKV reached a plateau at passage #2, whereas an increase in the production of CH-17-D/ZIKV* was observed from passage #6 to passage #8, reaching viral genome values similar to those observed with CH-17-D/ZIKV (2.67e+9 viral RNA copies/ml) (Fig. 2).

### CH-17-D/ZIKV genotypic characterization

To identify the genotypic determinants associated with the difference in viral replication observed between CH-17-D/ZIKV and CH-17-D/ZIKV*, the complete genome of CH-17-D/ZIKV was obtained at passages #2 and #4 and compared with the sequence of the original construct. Only five substitutions were detected at passage #2, of which two were non-synonymous, confirming the genome integrity of this strain (Table 1). In addition, four substitutions were already fixed or almost fixed. At passage #4, all these mutations were fixed, and no additional mutations were observed. Interestingly, both non-synonymous mutations are located in domain II of the E protein at residues E255 and E285^20^. Subsequently, we determined the sequence of the 5′ region of the CH-17-D/ZIKV* viral genome (until the NS1 coding region) at passage #4 and the complete genome sequence of CH-17-D/ZIKV* at passage #8 (Table 1). While only one transitory substitution was detected at passage #4, all the mutations that were detected in CH-17-D/ZIKV were detected at passage #8, including the two non-synonymous mutations located in the E coding region. This high level of parallel evolution associated with the observed chronology of events strongly suggests that these five mutations are associated with the increase in replicative fitness observed for both viruses.

**Table 1:** Mutations detected during the passages that followed cell transfection of chimeric viruses

Chimeric virus	Nucleotide position	Frequency at #P2	Frequency at #P4	Frequency at #P8	Frequency at #P10	Region	Nucleotide change	aa change
**CH-17-D/ZIKV**	291	100%	100%	100%	100%	C	A>G	–
	1625	66%	90%	92%	94%	E	T>C	V>A
	1706	100%	100%	100%	100%	E	G>T	G>V
	2514	100%	100%	100%	79%	NS1	A>G	–
	4482	100%	100%	100%	100%	NS2B	A>G	–
CH-17-D/ZIKV*	291	n.a	n.d	70%	n.a	C	A>G	–
	1303	n.a	100%	n.d	n.a	E	C>T	H>Y
	1625	n.a	n.d	68%	n.a	E	T>C	V>A
	1706	n.a	n.d	69%	n.a	E	G>T	G>V
	2514	n.a	n.d	69%	n.a	NS1	A>G	–
	4482	n.a	n.a	56%	n.a	NS2B	A>G	

Only consensus mutations (frequency >50%) are shown. *n.a.* not available, *n.d.* not detected

### CH-17-D/ZIKV initial characterization

**Fig. 3:**
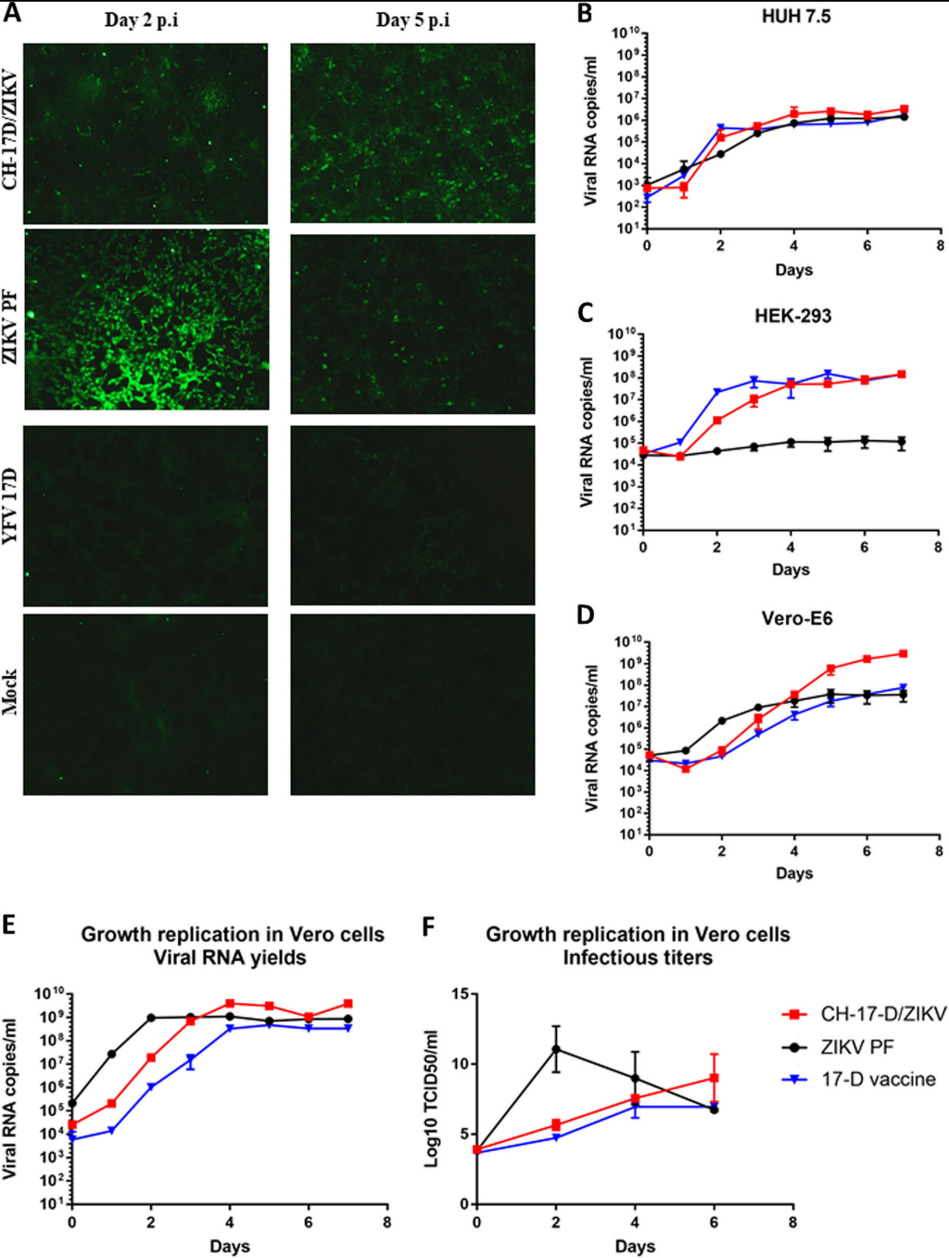
CH-17-D/ZIKV in cellulo characterization. **a** Expression of the ZIKV E protein in Vero-E6 was confirmed at day 2 and 5 post-infection using an indirect immunofluorescence assay with a specific ZIKV immune serum as the primary antibody. Uninfected cells (mock) and cells infected by ZIKV and the 17-D vaccine strain were used as controls. **b**–**d** Comparative growth kinetics of the CH-17-D/ZIKV and ZIKV 17-D vaccine strains in HUH7.5 (**b**), HEK-293 (**c**), and Vero-E6 cells (**d**). **e,** **f** Comparative growth kinetics of the CH-17-D/ZIKV and ZIKV 17-D vaccine strains in Vero cells. Cell supernatant medium was harvested at different time points after infection to assess the amount of viral RNA present using a real-time quantitative RT-PCR assay (**e**; expressed as the means ± SD) and the infectious titers using a TCID_50_ assay (**f**; expressed as the means ± SD)

To confirm the presence of the ZIKV E protein in Vero-E6 cells infected by the chimeric virus, we performed an indirect immunofluorescence assay using a specific ZIKV immune serum as the primary antibody (Fig. 3a). ZIKV PF and the 17-D vaccine strains were used as positive and negative controls, respectively. As expected, no fluorescence was observed with the 17-D vaccine strain, and positive cells were observed at day 2 and 5 post-infection with both the chimeric and ZIKV strains, confirming that the ZIKV E protein was expressed in infected cells. At day 2 post-infection, the number of cells that were positive for ZIKV was greater than that observed for CH-17-D/ZIKV, in agreement with the observed growth replication kinetics in Vero-E6 cells. Since a cytopathic effect was observed with the ZIKV strain at day 5, the number of positive cells was lower using this virus. Viability assays in Vero-E6 cells confirmed this observation; the CH-17-D/ZIKV virus is less cytopathic (mean value: 73% of cell viability) at day 5 post-infection than the ZIKV (mean value: 49% of cell viability) (Supplemental Fig. 1). We next performed comparative growth kinetics of these viruses in three different cell lines (HUH7.5, HEK-293, and Vero-E6). Cell supernatant medium was harvested at different time points after infection to assess the amount of viral RNA (Fig. 3b–d). Similar growth kinetics curves were observed for all viruses in HUH7.5 cells. In Vero-E6 cells, higher amounts of viral genome were not observed in cell supernatants until day 5 post-infection with the chimeric virus. In HEK-293 cells, the chimeric virus had a similar behavior to that of the 17-D vaccine strain.

### CH-17-D/ZIKV characterization in Vero cells

Since Vero cells are widely used for vaccine production^21^, we characterized CH-17-D/ZIKV in this cell line. Because CH-17-D/ZIKV was already adapted at passage #4 (see above), we used cell supernatant from this passage to perform growth kinetics in Vero cells. Cell supernatant medium was harvested at different time points after infection to assess infectious titers (via a TCID_50_ assay) and the amount of viral RNA (Fig. 3e, f). The results showed that these cells enabled the production of highly infectious viral particles at day 6 post-infection. We also studied the genetic stability of CH-17-D/ZIKV by performing six additional passages in Vero cells, and the complete genome sequence was obtained at passages #8 and #10 (Table 1). Our findings revealed a remarkable genetic stability since all mutations at passage #4 remained stable and no additional mutations were detected.

### CH-17-D/ZIKV in vivo characterization

Because ZIKV and the 17-D vaccine strain do not replicate in immunocompetent mice, we used immunocompromised mice as a model to study the chimeric virus in vivo. Each time animals were immunized or infected; they were transiently immunocompromised following a two-step inoculation with an anti-IFNAR antibody^22–24^ as described in the Methods section.

**Fig. 4:**
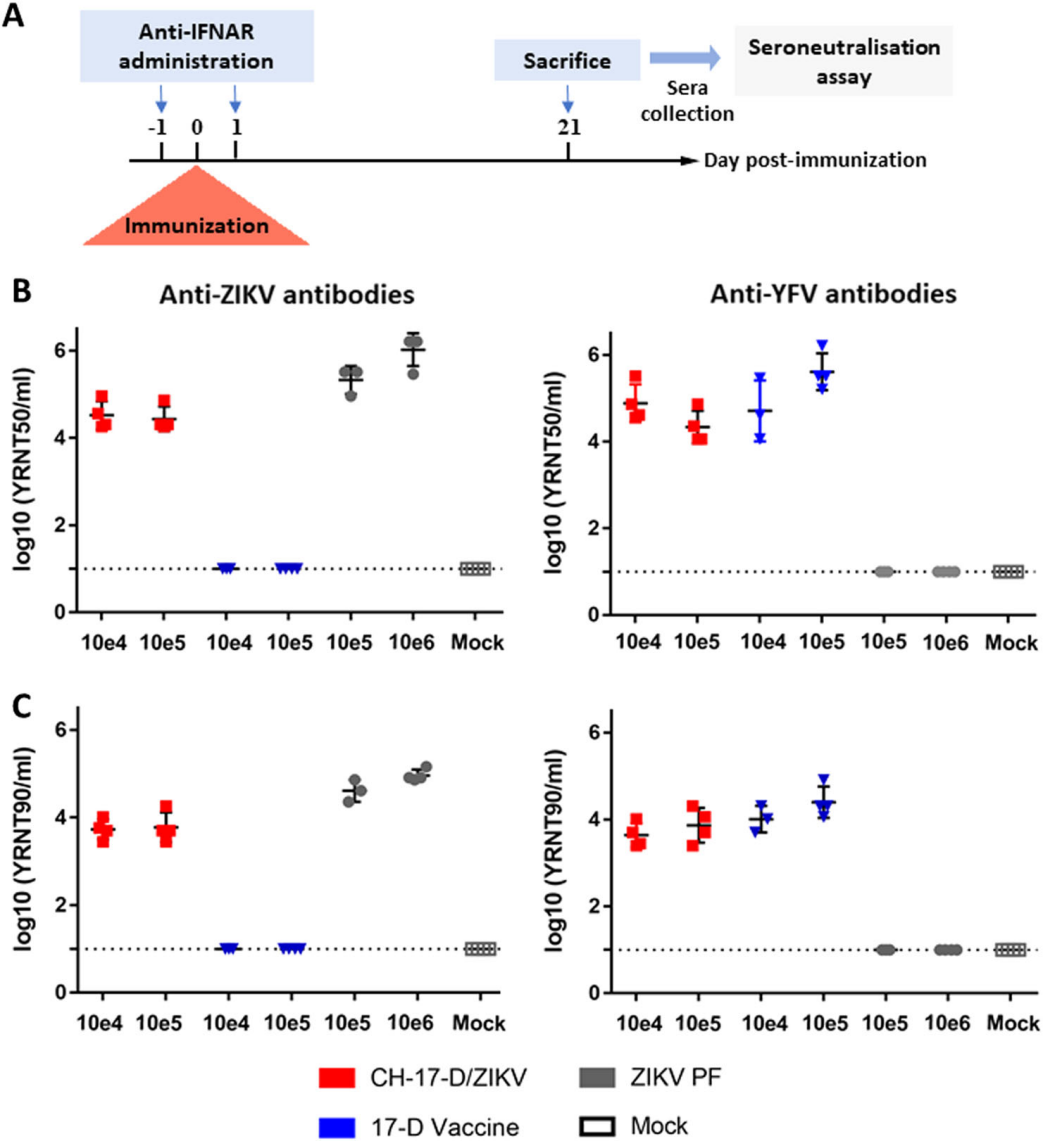
Neutralizing antibody titers in transitory immunocompromised mice at day 21 post-immunization. **a** Experimental timeline. **b**, **c** Groups of four mice were immunized with two doses of CH-17-D/ZIKV, ZIKV, and the 17-D vaccine strain (from 10e4 to 10e6 TCID_50_). Twenty-one days later, sera from mice were tested for the presence of antibodies to ZIKV and YFV using a viral RNA Yield Reduction Neutralization Test. The results are expressed as individual log of YRNT50 (**b**) and YRNT90 titers (**c**) with mean values ±SD represented by black lines with error bars, respectively

Six groups of four mice were inoculated with two different dosages of CH-17-D/ZIKV, ZIKV, or the 17-D vaccine strain to assess antibody production (Fig. 4a). A control group (mock) of four mice were inoculated with phosphate-buffered saline (PBS). Twenty-one days after immunization, mice were sacrificed and their sera were tested for the presence of antibodies to ZIKV and YFV (Fig. 4b, c) using a viral RNA Yield Reduction Neutralization Test (YRNT; see Methods). The results demonstrated that immunization with the chimeric virus induced the production of neutralizing antibodies against ZIKV, confirming the initial hypothesis of this study. We detected a slightly higher level of neutralizing antibodies when mice were infected with ZIKV. In all cases, both dosages used induced comparable neutralizing titers. Consistent with previous studies, mice immunized with the 17-D vaccine strain did not produce antibodies against ZIKV. Indeed, based on the amino acid sequence divergence of antigenic proteins, it is well established that no cross-neutralizing activity exists between these two distant flaviviruses^25^. As expected, mice immunized with the 17-D vaccine strain produced high levels of neutralizing antibodies against YFV, while those infected with ZIKV did not produce any antibodies against YFV. Interestingly, immunization with CH-17-D/ZIKV induced the production of neutralizing antibodies against YFV. This result demonstrated the immunogenicity of the viral proteins encoded by the 17-D vaccine strain backbone. We also attempted to isolate chimeric virus from animal blood samples to assess the ability of the chimeric virus to replicate in vivo. To avoid the possibility of isolating residual virus from the immunization, at days 2 and 3 post immunization we collected a blood drop from the tails of mice and found two positive samples (one from each day) (Supplemental Fig. 2). These findings suggest that CH-17-D/ZIKV is able to replicate in mice, since comparable neutralizing titers were measured with all mice immunized.

**Fig. 5:**
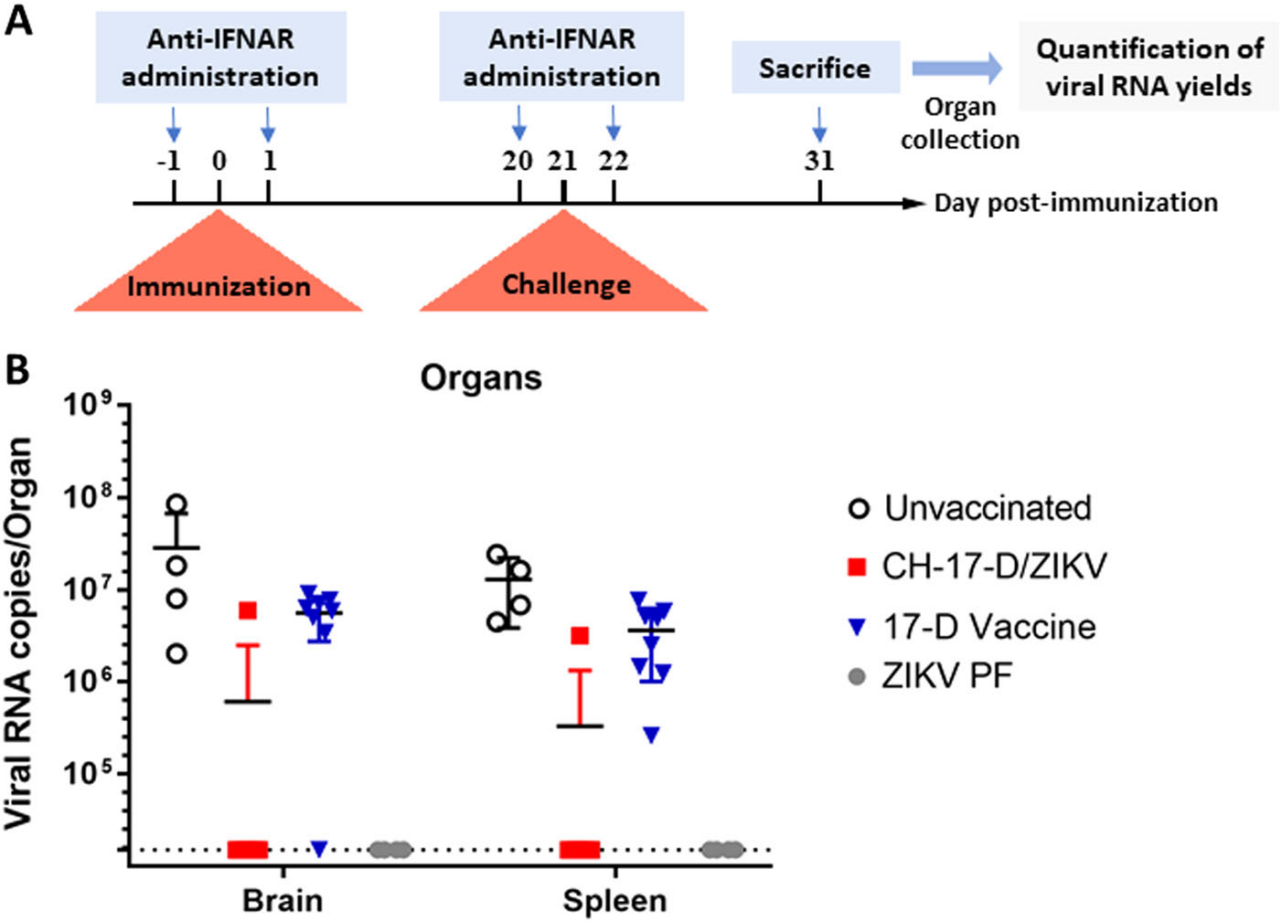
Amounts of viral RNA detected in brain and spleen samples collected during challenge experiments. **a** Experimental timeline. **b** Amounts of viral RNA in brain and spleen samples collected during challenge experiments (cf. Table 2) measured using a real-time quantitative RT-PCR assay. Mean values ± SD are represented by black lines and error bars, respectively. The results from both doses of viruses are pooled

In another experiment, we assessed protection against subsequent infection by wild-type ZIKV following immunization with CH-17-D/ZIKV or the 17-D vaccine strain (Fig. 5a). Groups of mice were immunized with two dosages of CH-17-D/ZIKV and the 17-D vaccine strain 21 days prior to challenge with a ZIKV African strain (Dak84). Three control groups were also used, one that was immunized with PBS and then challenged (the unvaccinated group), one that was immunized with ZIKV PF and then challenged (the ZIKV PF group), and one that was immunized and challenged with PBS (the mock group). Since 100% of the mice of the ZIKV PF control group were viremic at days 2 and 3 post-challenge, this criterion was not used to assess protection (Supplemental Fig. 3 and Supplemental Table 1). Therefore, the protection was evaluated by determining the proportion of mice with organs (brain and spleen) that tested positive for the presence of ZIKV at day 10 post-challenge. We observed that 10% of the spleens and brains from mice immunized with the chimeric virus (both groups) were positively tested positive for ZIKV (Table 2). In contrast, 100% and 87.5% of the spleens and brains from mice immunized with the 17-D vaccine strain (both groups) tested positive for ZIKV, respectively (*p*-value = 0.0004 for spleens and 0.0029 for brains; Fisher exact test). As expected 100% and 0% of the organs from mice in the unvaccinated group and from the ZIKV PF group were positive for ZIKV, respectively. Viral RNA yields from the organs were highly variable in all positive samples (Fig. 5b). These results demonstrated that immunization with the chimeric virus significantly protected mice against the systemic and brain infection induced by a heterologous ZIKV strain.

**Table 2:** Protection of transitory immunocompromised mice challenged with a heterologous strain of ZIKV

Viral strain	Spleens	Brains
CH-17D/ZIKV (both doses)	10% (1/10)	10% (1/10)
17-D vaccine strain (both doses)	100% (8/8)	87.5% (7/8)
Unvaccinated	100% (4/4)	100% (4/4)
ZIKV PF	0% (0/4)	0% (0/4)

Groups of mice were immunized with two doses (10e4 and 10e5 TCID_50_) of CH-17-D/ZIKV, the 17-D vaccine strain or PBS (unvaccinated). Twenty-one days later, mice were challenged with 10e6 TCID_50_ of an African ZIKV strain. The proportion of mice testing positive for ZIKV in spleen/brain samples at day 10 post-challenge was expressed as a percentage. The results from both doses of viruses are pooled (the results for individual groups are provided in Supplemental Table 2). Viral RNA was detected using a real-time RT-PCR assay. The amounts of viral RNA detected in samples are shown in Fig. 5

## Discussion

We present here the initial development of a chimeric ZIKV live-attenuated vaccine candidate based on a yellow fever attenuated 17-D genetic backbone. Using the ISA reverse genetics method, we were able to rapidly test several combinations of subgenomic amplicons starting from a pre-existing 17-D vaccine strain reverse genetics system. This method was recently applied to Asian and African strains of ZIKV^26^.

Three different designs were tested to incorporate the prM/E of ZIKV into the 17-D vaccine genetic backbone. Our results highlighted the necessity of modifying the cleavage site between the pre-peptide and the prM protein for the construction of chimeric viruses, as was previously described during development of chimeric ZIKV/DENV and DENV/ZIKV strains^27^.

Nevertheless, we also demonstrated that chimeric viruses are needed to acquire adaptive mutations to properly replicate in mammalian cells. Indeed, we observed a low percentage of virus recovery during cell transfection experiments, and both replicative viruses rescued shared five substitutions, of which two were non-synonymous and located in domain II of the E protein. Interestingly, mutations located in this particular domain of the E protein were previously described in cellulo with 17-D vaccine strain-based chimeric flaviviruses (DENV type 1/2 and Japanese encephalitis virus)^28,29^. These findings suggest that the emergence of compensatory mutations in the E protein is probably necessary to restore the replicative fitness of the virus following the exchange of two of its structural proteins.

By comparing the growth properties of our chimeric virus with its two parental strains in different mammalian cells, we observed that this new synthetic virus had its own biological properties, probably due to the nature of this new combination of viral proteins. In fact, we observed than this strain is fitter than parental strains in Vero-E6 cells and is close to the fitness of the 17-D vaccine strain in HEK-293 cells.

Genetic stability is a major concern when designing future live-attenuated vaccine candidates. Using Vero cells, which are widely used for vaccine production^21^, and the adapted chimeric virus, we performed serial passages to assess this essential criterion. We demonstrated that once initial adaptation was achieved, the chimeric virus remained genetically stable.

We used transitory immunocompromised mice as an animal model system to characterize the chimeric virus in vivo. We demonstrated that mice infected with this virus produced levels of neutralizing antibodies that were close to those observed following infection by ZIKV. Our results also showed that immunization using the chimeric strain significantly protected 90% of mice against brain and spleen invasion induced by a heterologous strain of ZIKV. This incomplete protection (i.e., one mouse out of ten was unprotected) could result from the incomplete CD8+ T cell immunity induced by structural proteins alone^30–32^. This explanation has been recently proposed to explain the failure of the CYD-TDV dengue chimeric vaccine^33^. Altogether, these results provide evidence that this chimeric strain has all the prerequisites needed to be tested in a more relevant animal model, such as the microcephalic-sensitive mouse model^34^.

The strategy used in the present study to develop a live ZIKV vaccine candidate has several advantages, including that the 17-D vaccine strain has long history of use in hundreds of millions of persons^35^ and is considered as the safest live-attenuated vaccine^36^. Moreover, compared with targeted attenuation strategies, such as local modification of genomic regions, our approach eliminates the risk of phenotype reversion by potential homologous recombination. Finally, although the potential occurrence of the antibody-dependent enhancement phenomenon has to be considered with chimeric vaccines, there is currently no epidemiological data supporting this hypothesis in areas where several flaviviruses co-circulate^37^.

In conclusion, our data provide a sound basis for the future development of this vaccine candidate. Furthermore, the approach used in this study to rescue the chimeric virus showed that significant advances in the development of reverse genetics methods now offer the possibility of drastically reducing the time frame between the emergence of a novel viral pathogen and the availability of a live-attenuated vaccine candidate.

## Materials and methods

### Cell lines

All cells were grown at 37 °C with 5 % CO_2_ with 1 % penicillin/streptomycin (PS; 5000 U/ml and 5000 µg/ml, respectively; Life Technologies) and supplemented with 1% non-essential amino acids (Life Technologies) in media as specified below.

Baby hamster kidney (BHK-21; ATCC number CCL-10), human hepatocellular carcinoma (HUH7.5^38^; RRID CVCL_7927) and human embryonic kidney (HEK-293; ATCC number CCL-1573) cells were grown in Dulbecco’s modified Eagle’s medium high glucose (4500 mg/l) (Life Technologies) with 7 .5% heat-inactivated fetal bovine serum (FBS; Life Technologies). Vero (ATCC number CCL-81) and Vero-E6 (ATCC number CRL-1586) cells were grown in minimal essential medium (Life Technologies) with 7 .5% FBS.

### Viruses

ZIKV Asian lineage strains PF (H/PF/2013, GenBank accession number: KJ776791) and Mart2015 (MRS_OPY_Martinique_PaRi_2015, GenBank accession number: KU647676), ZIKV African lineage strain Dak84 (A.taylori-tc/SEN/1984/41662-DAK, GenBank accession number: KU955592), YFV 17-D strain (produced by reverse genetics as described below; GenBank accession number: EU074025), and YFV strain BOL 88/1999 (isolated in 2009 from human serum and kindly provided by the National Center of Tropical Diseases (CENETROP), Santa-Cruz, Bolivia, GenBank accession number: KF907504) were used in this study. All these viral strains are available for the scientific community via the European Virus Archive goes Global (EVAg) project, a non-profit organization (https://www.european-virus-archive.com).

For each viral strain, we prepared a stock solution of clarified cell culture medium that was subsequently used for all analyses. Briefly, a 25 cm^2^ culture flask of confluent Vero-E6 cells containing 667 µl of medium with 2.5% FBS (Life Technologies) was inoculated with 333 µl of clarified infectious medium, incubated for 6 h, washed once with Hank’s Balanced Salt Solution (HBSS, Life Technologies), and then incubated for 3 days with 7 ml of fresh medium. Cell supernatant medium was subsequently harvested and clarified by centrifugation, supplemented with HEPES buffer (final concentration of 25 mM; Sigma), and then aliquoted and stored at −80 °C.

All experiments using replicating viruses were performed in BSL3 facilities.

### ISA procedure

Chimeric viruses and the YFV 17-D vaccine strain were rescued using the ISA (Infectious Subgenomic Amplicons) reverse genetics method as previously described^19,26,39,40^.

#### Preparation of subgenomic DNA fragments

The complete viral genome was amplified by PCR as three overlapping DNA fragments. The first and last fragments were flanked by the 5′ and 3′ termini, which included the human cytomegalovirus promoter (pCMV) and the hepatitis delta ribozyme followed by the simian virus 40 polyadenylation signal (HDR/SV40pA), respectively. We started by using a reverse genetics system designed for the YFV 17-D strain (described in the Supplemental Material). Because the first DNA fragment contained all the regions encoding structural genes, only this fragment was modified to design chimeric viruses (the primers are listed in Supplemental Table 3).

DNA fragments were generated by PCR using de novo synthesized genes (Genscript) as templates. The sequences of the primers used are listed in Supplemental Table 2. PCR mixes were prepared using a Platinum PCR SuperMix High Fidelity kit (Life Technologies) following the manufacturer’s instructions. PCR amplifications were performed using an ABI 2720 thermal cycler (Applied Biosytems) with the following conditions: 94 °C for 2 min followed by 40 cycles of 94 °C for 15 s, 60 °C for 30 s, 68 °C for 5 min, with a 10 min final elongation at 68 °C. PCR product sizes and quality were controlled by running gel electrophoresis and DNA fragments were purified using a High Pure PCR Product Purification kit (Roche).

#### Cell transfection

Mixtures of BHK-21 and HEK-293 cells were seeded into PureCoat amine six-well cell culture plates (Corning) 1 day prior to transfection. Cells were transfected with 2 µg of an equimolar mix of the three DNA fragments using lipofectamine 3000 (Life Technologies) following the manufacturer’s instructions. Each transfection was performed in five replicates. After incubating for 24 h, the cell supernatant medium was removed and replaced by fresh cell culture medium. Six days post-transfection, cell supernatant medium was passaged four times using six-well cell culture plates of confluent Vero-E6 cells. Cells were subsequently inoculated with 100 µl of diluted (1/3) cell supernatant media, incubated 2 h, washed with HBSS, and incubated 6 days with 3 ml of medium. Remaining cell supernatant medium was stored at −80 °C and samples were referred to as passages #1, #2, #3, and #4. To ensure the complete removal of DNA used during the transfection, passage #4 was used to assess viral replication, where 100 µl of cell supernatant medium was collected to detect viral RNA using a qRT-PCR assay (see below). Passage #3 was used to produce virus stock solutions of YFV 17-D and chimeric viruses.

### RNA extraction and real-time quantitative PCR assays

RNA extraction was performed using the Qiacube HT and the Cador pathogen extraction kits (both from Qiagen) following the manufacturer’s instructions. Briefly, 100 µl of cell supernatant medium was transferred into an S-block containing the recommended quantities of VXL, proteinase K and RNA carrier. A DNAse digestion step (Qiagen) was performed to remove the DNA used during cell transfection. The quantity of viral RNA was quantified by real-time quantitative RT-PCR (qRT-PCR; EXPRESS One-Step Superscript™ qRT-PCR Kit, universal; Life Technologies). The sequences of the primers used to detect ZIKVs, YFV 17-D, and chimeric viruses are listed in Supplemental Table 4. For each reaction, 3.5 µl of RNA was used (final volume of 10 µl) and amplifications were performed using a QuantStudio 12 K Flex Real-Time PCR System (Applied Biosytems) with the following conditions: 10 min at 50 °C and 2 min at 95 °C, followed by 40 amplification cycles (95 °C for 3 s followed by 30 s at 60 °C). The amounts of viral RNA present were calculated from standard curves (quantified T7-generated synthetic RNA standards were used).

### Complete genome sequencing

Complete and partial genome sequencing of chimeric viruses were performed as previously described^40^. Viral RNA extraction was performed as described above. A set of specific primer pairs (Supplemental Table 5) was used to generate amplicons by RT-PCR using a Superscript III One-Step RT-PCR Platinum TaqHifi kit (Life Technologies). For each passage sequenced, purified PCR products were pooled and analyzed using an Ion PGM Sequencer (Life Technologies) according to the manufacturer’s instructions. The resulting reads were analyzed using CLC Genomics Workbench 6 (CLC Bio). The sequences were trimmed based on quality scores by removing the primer sequences at their termini and systematically removing 20 nt at the 5′ and 3′ termini. The remaining reads with lengths greater than 99 nt were mapped using the designed sequence of the chimeric virus as a reference to obtain a consensus sequence. The mutation frequency for each position was calculated as the number of mutated reads divided by the total number of reads at that site.

### Tissue culture infectious dose 50 (TCID_50_) assay

A 96-well cell culture plate containing confluent Vero-E6 cells with 100 µl/well of media were inoculated with 10-fold serial dilutions of centrifugation-clarified cell culture supernatant medium (50 µl/well). Each dilution was repeated six times. The plate was incubated for 7 days and read to assess the absence or presence of CPE in each well. TCID_50_ titers were subsequently calculated using the Reed–Muench method^41^.

### Cell viability assay

Confluent cells were inoculated at an MOI (multiplicity of infection) of 0.01 in a 96-well cell culture plate in triplicate for each measurement. Every day for a period of 5 days we performed the cell titer blue viability assay (Promega) following the manufacturer’s instructions.

### Virus growth kinetics

Confluent cells were inoculated at an MOI of 0.01 in a six-well cell culture plates in triplicate. Every day for a period of 7 days, 100 µl of cell supernatant medium was collected to measure the amount of viral RNA by qRT-PCR (see above) and 200 µl was collected to assess TCID_50_ values.

### Indirect immunofluorescence assay

Confluent Vero-E6 cells were inoculated at an MOI of 0.01 in an eight-well cell culture Lab-Tek II Chamber Slide System in duplicate. At 2 and 5 days post-infection, cells were washed twice with HBSS and fixed with 4% paraformaldehyde for 2 h. Viral antigens were detected as previously described^19,42^ using a specific ZIKV immune serum as the primary antibody (dilution: 1/50) collected from a Syrian Hamster immunized with the ZIKV strain Mart2015 (see below). This serum was shown to neutralize more than 90% of ZIKV PF replication up to a 1/3000 dilution (data not shown). The secondary antibody used was a goat anti-hamster Alexa 488 antibody (Invitrogen), which was used at a 1/500 dilution. Slides were observed using an Eurostar II fluorescence microscope with the Europicture software (Euroimmune).

### Viral RNA YRNT

Vero-E6 cells were seeded into a 96-well cell culture plate 1 day prior to infection (5 × 10^4^ cells in 100 µl of medium containing 2.5% FBS per well). The next day, two-fold serial dilutions of sera (from 1/20 to 1/2560; diluted with medium containing 2.5% FBS) were mixed (50:50; v/v) with appropriate amounts of viral stock (diluted in medium containing 2.5% FBS), incubated for 1.5 h at 37 °C under a 5% CO_2_ atmosphere, and then were added to cells (50 µl/well). The amount of virus added had been calibrated to ensure that virus production in the cell supernatant medium did not reach a plateau at the readout time^43^. Cells were incubated for 3 days, after which 100 µl of cell supernatant medium was harvested to perform nucleic acid extraction and to quantify amounts of viral RNA using a qRT-PCR assay (see above). Each serum dilution was tested in triplicate and duplicate for the control group. For each experiment, a virus replication control (VC) was performed in quadruplicate to assess viral replication. For each serum dilution, viral RNA yield reduction (% of viral inhibition) was calculated using the mean amount of viral RNA obtained with VC as a reference. The 50% and 90% viral inhibition cut-offs were used to estimate viral RNA Yield Reduction Neutralization 50% and 90% (YRNT50; YRNT90) titers using the method of Reed and Muench^41^.

### In vivo experiments

#### Animal handling

Animals were maintained in an ISOcage P Bioexclusion System (Techniplast) with unlimited access to food and water and 12 h light/12 h dark cycle. Animals were individually monitored every day to detect the appearance of any clinical sign of illness/suffering. Virus/Antibody inoculation, blood collection, and euthanasia (cervical dislocation) were performed under general anesthesia (isofluorane).

#### Golden hamster immunization

One 4-week-old female Syrian Hamster (Janvier) was intraperitoneally immunized with 100 μl containing 10^5^ TCID_50_ of ZIKV strain Mart2015. After 21 days, the Hamster was re-injected with the same dose. The hamster did not show any sign of illness or weight loss. After an additional 15 days, the hamster was euthanized and a blood sample (intracardiac puncture) was collected. After centrifugation, the serum was stored at −80 °C.

#### Administration of anti-IFNAR antibody

All the mice used were immunocompromised following a two-step inoculation of an anti-IFNAR antibody (clone MAR1-5A3; Interchim; intraperitoneal injection; 120 µl)^22,24^, with 1 mg administered 1 day prior and 1 day after each infection/immunization (i.e., the mice challenged were immunocompromised twice with this two-step procedure).

#### Mouse immunization

Six groups of four 3-week-old female C57/bl6 mice (Charles River) were intraperitoneally inoculated with 100 µl of virus: two groups were immunized with the YFV 17-D strain (two dosages: 10^4^ and 10^5^ TCID_50_), two groups were immunized with the ZIKV PF strain (two dosages: 10^5^ and 10^6^ TCID_50_), and two groups were immunized with the CH-17-D/ZIKV strain (two dosages: 10^4^ and 10^5^ TCID_50_). A control group of four mice was used as a negative control group (non-immunized mice).

Blood collection (10 µl) from the tail vein was performed at days 2 and 3 post-immunization to detect infectious virus by cell culture isolation. Immediately after collection, all the blood was inoculated into a 12-well cell culture plate containing confluent Vero-E6 cells and 150 µl of medium/well. After incubating for 2 h, 100 µl of the inoculum was harvested. The cells were washed with HBSS and then 1.5 ml/well of fresh medium was added to the cells, which were incubated for 6 days. Finally, 100 µl of cell supernatant medium was harvested to perform nucleic acid extraction and to quantify amounts of viral RNA using a real-time qRT-PCR assay as described above.

At day 21 post-infection, all animals were euthanized and blood samples were collected via intracardiac puncture. After the blood samples were centrifuged, sera were stored at −80 °C before being used to perform the neutralization tests.

#### Challenge experiments

Five groups of four 3-week-old female C57/bl6 mice (Charles River) were intraperitoneally inoculated with 100 µl of virus: two groups were immunized with the YFV 17-D strain (two dosages: 10^4^ and 10^5^ TCID_50_), two groups were immunized with the CH-17-D/ZIKV strain (two dosages: 10^4^ and 10^5^ TCID_50_), and one group was immunized with the ZIKV PF strain (10^5^ TCID_50_). Two control groups of four mice were used as a (i) mock control group (non-immunized/non-challenged mice) and (ii) a negative control group (non-immunized mice; challenged).

All animals (except the mock control group) were then challenged with 10^6^ TCID_50_ of ZIKV Dak84. Blood collection (10 µl) from the tail vein was performed at days 2 and 3 post-challenge to assess viremia by qRT-PCR. At day 10 post-challenge, all the animals were euthanized. Organs (spleen and brain) were then collected in 1 ml of HBSS supplemented with 10% of FBS and crushed for 10 min at 30 cycles per second with tungsten beads using a Tissue Lyser machine (Retsch MM400). After centrifugation at 5000 *g* for 10 min, the supernatant medium was collected and then centrifuged again at 10,000 *g* for 10 min. Fifty microliters of the supernatant medium was used to perform nucleic acid extraction and to quantify the amount of viral RNA using a real-time qRT-PCR assay (see above).

### Statistical analysis

All data obtained were analyzed using Graphpad Prism 7 (Graphpad software), which was also used for all graphical representations and statistical analyses.

## Electronic supplementary material

Supplemental material
