# Supplementary material for: Live Zika virus chimeric vaccine candidate based on a yellow fever 17-D attenuated backbone
Source: Emerg Microbes Infect. 2018 Sep 26;7:161. doi: 10.1038/s41426-018-0161-7 (PMC6156337; doi:10.1038/s41426-018-0161-7)
Supplement: Supplementary file 1 — Supplemental material [file 41426_2018_161_MOESM1_ESM.docx]

**Supplemental data**

# **Live Zika virus chimeric vaccine candidate based on a yellow fever 17-D attenuated backbone**

Franck Touret, Magali Gilles, Raphaelle Klitting, Fabien Aubry, Xavier de-Lamballerie and Antoine Nougairède


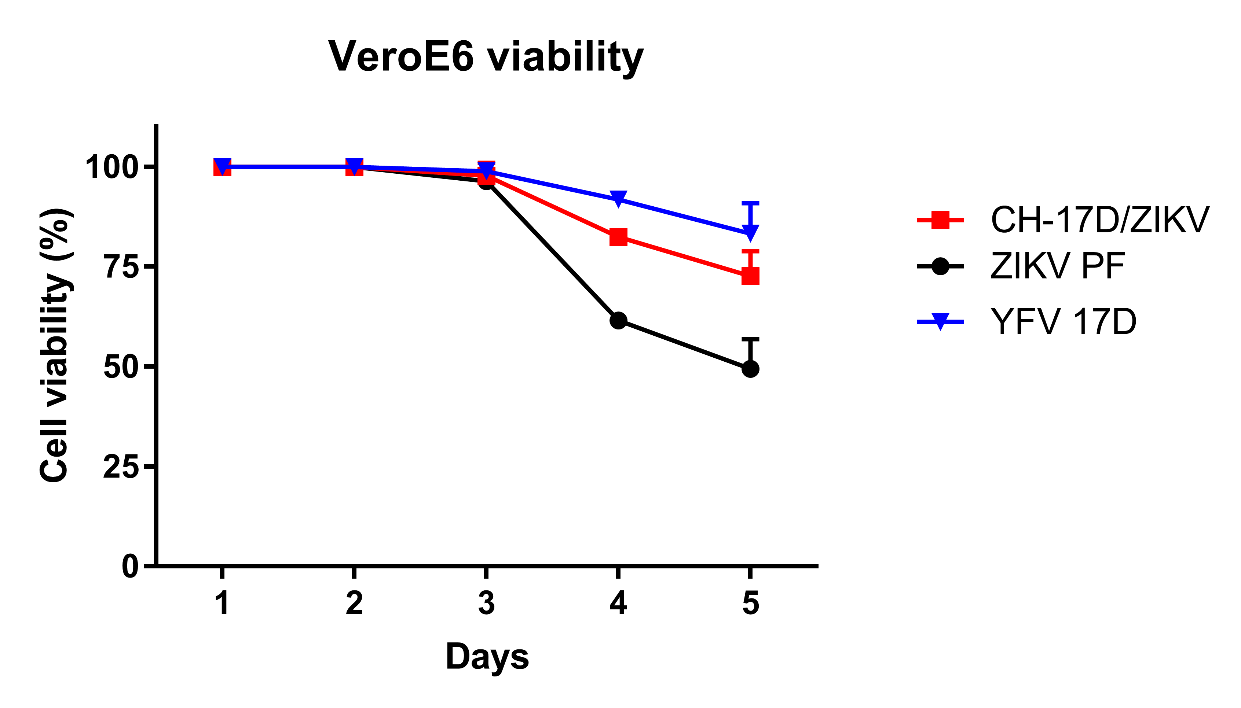


**Supplemental figure 1 :** **Cell viability assay performed in Vero-E6** **cells**

Cells were infected with CH-17-D/ZIKV, ZIKV and the 17-D vaccine strains (viability is expressed as percentage with mean ±SD).


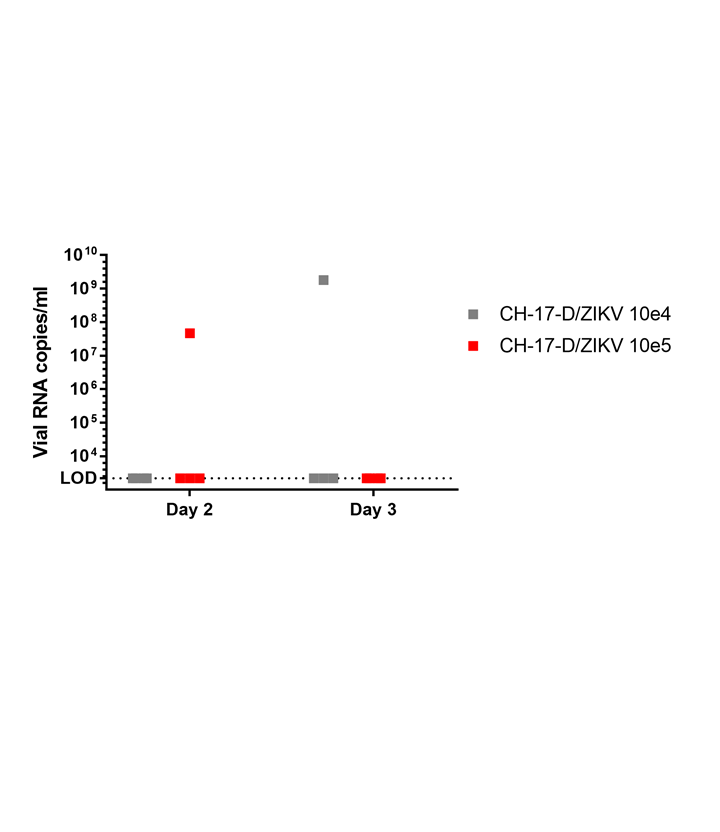


**Supplemental figure 2: Detection of infectious virus using blood of mice collected at day 2 and 3 post-immunization**

Detection of infectious virus was performed by cell culture isolation. Blood was inoculated into a 12-well cell culture plate of Vero-E6 cells. After an incubation period of 6 days, 100µL of cell supernatant medium was harvested to perform nucleic acid extraction and to quantify amounts of viral RNA using a real-time qRT-PCR assay.

**Supplemental table 1: Viremia of transitory immunocompromised mice challenged with a heterologous strain of ZIKV**

| **Viral strain** | **Viremia day 2** | **Viremia day 3** |
| --- | --- | --- |
| **CH-17D/ZIKV**  **(both doses)** | **30% (3/10)** | **60 % (6/10)** |
| **17-D vaccine strain**  **(both doses)** | **75 % (6/8)** | **100 % (8/8)** |
| **Unvaccinated** | **100 % (2/2)** | **100 % (4/4)** |
| **ZIKV PF** | **100% (4/4)** | **100% (4/4)** |

Groups of mice were immunized with two doses (10e4 and 10e5 TCID_50_) of CH-17-D/ZIKV, the 17-D vaccine strain or PBS (unvaccinated). Twenty-one days later, mice were challenged with 10e6 TCID_50_ of a ZIKV African strain. The proportion of mice with detectable viremia at days 2 and 3 post-challenge was expressed as percentage. Results from both doses of viruses are pooled (results for each group are in supplemental Table 2). Detection of viral RNA was assessed using a real time RT-PCR assay.

**
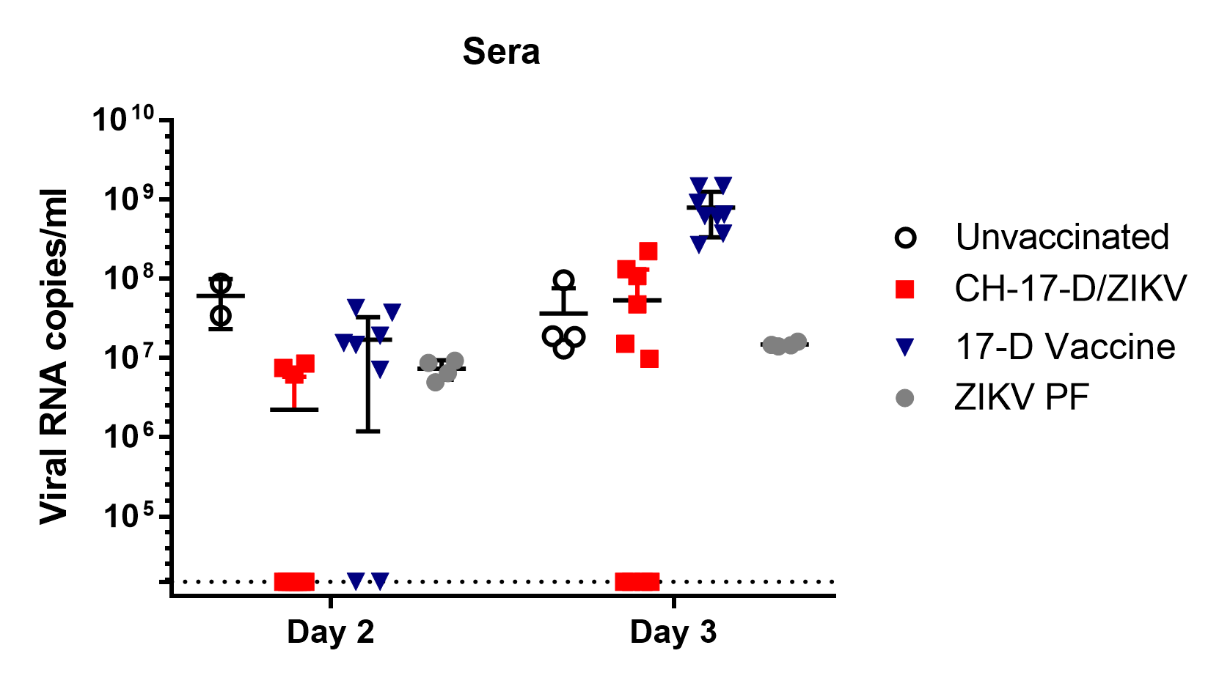
 Supplemental figure 3: Amounts of viral RNA detected in blood collected during challenge experiments**

Amounts of viral RNA in blood collected during challenge experiments measured using a real time quantitative RTPCR assay. Mean values ±SD are represented respectively by black lines and error bars.

**Supplemental table 2: Protection of transitory immunocompromised mice challenged with a heterologous strain of ZIKV**

The mice were distributed into groups of four. Each group was immunized with either 10e4 or 10e5 TCID_50_ of CH17D/ZIKV, the 17-D vaccine strain. Three control groups were also used: one immunized with PBS and then challenged (unvaccinated), one immunized with 10e5 TCID50 of ZIKV PF and then challenged (ZIKV PF) and one immunized and challenged with PBS (mock). Twenty-one days later, mice were challenged intraperitoneally, with 10e6 TCID_50_ of a ZIKV African strain. The proportion of mice with detectable viremia at day 2 and 3 post challenge and with positive spleen/brain at day 10 post challenge was expressed as percentage. Detection of viral RNA was assessed using a real time RTPCR assay. Amounts of viral RNA detected in positive samples are represented in Figure B in S1 Text).

| **Viral strain** | **Viremia day 2** | **Viremia day 3** | **Spleens** | **Brains** |
| --- | --- | --- | --- | --- |
| **CH17D/ZIKV (10e4)** | 33% (2/6) | 66% (4/6) | 16 % (1/6) | 16 % (1/6) |
| **CH17D/ZIKV (10e5)** | 25 % (1/4) | 50% (2/4) | 0% (0/4) | 0% (0/4) |
| **17-D vaccine strain (10e4)** | 75% (3/4) | 100% (4/4) | 100% (4/4) | 75% (3/4) |
| **17-D vaccine strain (10e5)** | 75% (3/4) | 100% (4/4) | 100% (4/4) | 100% (4/4) |
| **ZIKV PF 10e5** | 100% (4/4) | 100% (4/4) | 0% (0/4) | 0% (0/4) |
| **Unvaccinated** | 100 % (2/2) | 100 % (4/4) | 100 % (4/4) | 100 % (4/4) |


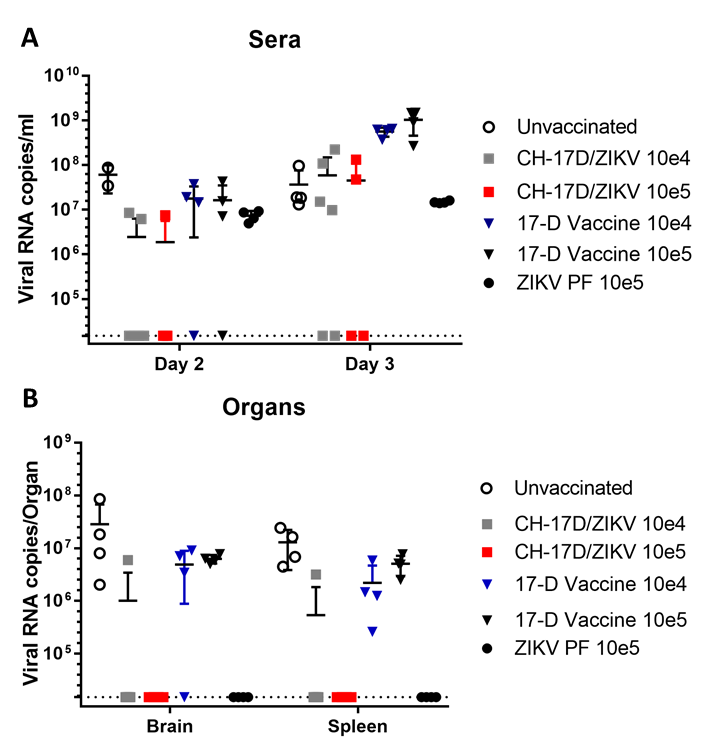
**Supplemental figure 4: Amounts of viral RNA detected in positive blood, brain and spleen samples collected during challenge experiments**

Amounts of viral RNA in sera (**A**) and organs (**B**) collected during challenge experiments measured using a real time quantitative RTPCR assay. Mean values ±SD are represented respectively by black lines and error bars.

**Supplemental Table 3: Primers used to generate subgenomic DNA fragments**

| **pCMV_F** | CACCCAACTGATCTTCAGCATCT | Fragment I |
| --- | --- | --- |
| **17D_I_Rint** | TGGATCCTGCACGACAACAG |  |
| **17D_II_Fint** | TTGAGCATGAGATGTGGAGAAG | Fragment II |
| **17D_II_Rint** | CCATCAACCACAGGGTTCTTG |  |
| **17D_III_Fint** | TTACCTGGAATCAAAGCGCAG | Fragment III |
| **HDR/SV40pA_R** | CTCAGGGTCAATGCCAGCGCTT |  |

**Supplemental Table 4: Primers used for real-time quantitative RT-PCR assays**

Each qRT-PCR was used with one or two viral strains (indicated in the last column). The region of the viral genome targeted by each system is under parentheses in the last column.

| **17DF** | TCCACTCATGAAATGTACTACGTGTCT | 17D vaccine strain  (NS5) |
| --- | --- | --- |
| **17DP** | FAM AGCCCGCAGCAATGTCACATTTACTGT MGB |  |
| **17DR** | GGAGGCGGGATGTTTGGT |  |
| **ZikaF2** | AARGACGGGAGRTCCATTGTG | ZIKV Dak84  (NS5) |
| **ZikaR2** | GRGCYCGGCCAATCAG |  |
| **ZikaP2** | FAM CGCCACCAAGATGA MGB |  |
| **ZikaEnv_P** | FAM AGCTGCCATCGCTT MGB | ZIKV PF / Chimeric viruses  (E) |
| **ZikaENV_F** | CCTGGCTTCGCGTTAGCA |  |
| **ZikaENV_R** | GCTCGTTGAGCTTCCCAAAA |  |

**Supplemental Table 5: Primers used for complete genome sequencing**

| **17D_IS0_F** | ATGTCTGGTCGTAAAGCTCAG | PCR product 1 |
| --- | --- | --- |
| **17D_I_Rint** | TGGATCCTGCACGACAACAG |  |
| **17D_II_Fint** | TTGAGCATGAGATGTGGAGAAG | PCR product 2 |
| **17D_II_Rint** | CCATCAACCACAGGGTTCTTG |  |
| **17D_III_Fint** | TTACCTGGAATCAAAGCGCAG | PCR product 3 |
| **17D_III_R** | AGTGGTTTTGTGTTTGTCATCC |  |

**Sequences of the subgenomic DNA fragments used during the ISA procedure**

Fragment I pCMV_CH17D/ZIKV construct A

CACCCAACTGATCTTCAGCATCTTCAATATTGGCCATTAGCCATATTATTCATTGGTTATATAGCATAAATCAATATTGGCTATTGGCCATTGCATACGTTGTATCTATATCATAATATGTACATTTATATTGGCTCATGTCCAATATGACCGCCATGTTGGCATTGATTATTGACTAGTTATTAATAGTAATCAATTACGGGGTCATTAGTTCATAGCCCATATATGGAGTTCCGCGTTACATAACTTACGGTAAATGGCCCGCCTGGCTGACCGCCCAACGACCCCCGCCCATTGACGTCAATAATGACGTATGTTCCCATAGTAACGCCAATAGGGACTTTCCATTGACGTCAATGGGTGGAGTATTTACGGTAAACTGCCCACTTGGCAGTACATCAAGTGTATCATATGCCAAGTCCGCCCCCTATTGACGTCAATGACGGTAAATGGCCCGCCTGGCATTATGCCCAGTACATGACCTTACGGGACTTTCCTACTTGGCAGTACATCTACGTATTAGTCATCGCTATTACCATGGTGATGCGGTTTTGGCAGTACACCAATGGGCGTGGATAGCGGTTTGACTCACGGGGATTTCCAAGTCTCCACCCCATTGACGTCAATGGGAGTTTGTTTTGGCACCAAAATCAACGGGACTTTCCAAAATGTCGTAATAACCCCGCCCCGTTGACGCAAATGGGCGGTAGGCGTGTACGGTGGGAGGTCTATATAAGCAGAGCTCGTTTAGTGAACCGAGTAAATCCTGTGTGCTAATTGAGGTGCATTGGTCTGCAAATCGAGTTGCTAGGCAATAAACACATTTGGATTAATTTTAATCGTTCGTTGAGCGATTAGCAGAGAACTGACCAGAACATGTCTGGTCGTAAAGCTCAGGGAAAAACCCTGGGCGTCAATATGGTACGACGAGGAGTTCGCTCCTTGTCAAACAAAATAAAACAAAAAACAAAACAAATTGGAAACAGACCTGGACCTTCAAGAGGTGTTCAAGGATTTATCTTTTTCTTTTTGTTCAACATTTTGACTGGAAAAAAGATCACAGCCCACCTAAAGAGGTTGTGGAAAATGCTGGACCCAAGACAAGGCTTGGCTGTTCTAAGGAAAGTCAAGAGAGTGGTGGCCAGTTTGATGAGAGGATTGTCCTCAAGGAAACGCCGTTCCCATGATGTTCTGACTGTGCAATTCCTAATTTTGGGAATGCTGTTGATGACGGGTGGAGTGACCTTGGTGCGGAAAGGGAGTGCATACTATATGTACTTGGACAGAAACGACGCTGGGGAGGCCATATCTTTTCCAACCACATTGGGGATGAATAAGTGTTATATACAGATCATGGATCTTGGACACATGTGTGATGCCACCATGAGCTATGAATGCCCTATGCTGGATGAGGGGGTGGAACCAGATGACGTCGATTGTTGGTGCAACACGACGTCAACTTGGGTTGTGTACGGAACCTGCCATCACAAAAAAGGTGAAGCACGGAGATCTAGAAGAGCTGTGACGCTCCCCTCCCATTCCACTAGGAAGCTGCAAACGCGGTCGCAAACCTGGTTGGAATCAAGAGAATACACAAAGCACTTGATTAGAGTCGAAAATTGGATATTCAGGAACCCTGGCTTCGCGTTAGCAGCAGCTGCCATCGCTTGGCTTTTGGGAAGCTCAACGAGCCAAAAAGTCATATACTTGGTCATGATACTGCTGATTGCCCCGGCATACAGCATCAGGTGCATAGGAGTCAGCAATAGGGACTTTGTGGAAGGTATGTCAGGTGGGACTTGGGTTGATGTTGTCTTGGAACATGGAGGTTGTGTCACCGTAATGGCACAGGACAAACCGACTGTCGACATAGAGCTGGTTACAACAACAGTCAGCAACATGGCGGAGGTAAGATCCTACTGCTATGAGGCATCAATATCGGACATGGCTTCGGACAGCCGCTGCCCAACACAAGGTGAAGCCTACCTTGACAAGCAATCAGACACTCAATATGTCTGCAAAAGAACGTTAGTGGACAGAGGCTGGGGAAATGGATGTGGACTTTTTGGCAAAGGGAGCCTGGTGACATGCGCTAAGTTTGCATGCTCCAAGAAAATGACCGGGAAGAGCATCCAGCCAGAGAATCTGGAGTACCGGATAATGCTGTCAGTTCATGGCTCCCAGCACAGTGGGATGATCGTTAATGACACAGGACATGAAACTGATGAGAATAGAGCGAAGGTTGAGATAACGCCCAATTCACCAAGAGCCGAAGCCACCCTGGGGGGTTTTGGAAGCCTAGGACTTGATTGTGAACCGAGGACAGGCCTTGACTTTTCAGATTTGTATTACTTGACTATGAATAACAAGCACTGGTTGGTTCACAAGGAGTGGTTCCACGACATTCCATTACCTTGGCACGCTGGGGCAGACACCGGAACTCCACACTGGAACAACAAAGAAGCACTGGTAGAGTTCAAGGACGCACATGCCAAAAGGCAAACTGTCGTGGTTCTAGGGAGTCAAGAAGGAGCAGTTCACACGGCCCTTGCTGGAGCTCTGGAGGCTGAGATGGATGGTGCAAAGGGAAGGCTGTCCTCTGGCCACTTGAAATGTCGCCTGAAAATGGATAAACTTAGATTGAAGGGCGTGTCATACTCCTTGTGTACCGCAGCGTTCACATTCACCAAGATCCCGGCTGAAACACTGCACGGGACAGTCACAGTGGAGGTACAGTACGCAGGGACAGATGGACCTTGCAAGGTTCCAGCTCAGATGGCGGTGGACATGCAAACTCTGACCCCAGTTGGGAGGTTGATAACCGCTAACCCCGTAATCACTGAAAGCACTGAGAACTCTAAGATGATGCTGGAACTTGATCCACCATTTGGGGACTCTTACATTGTCATAGGAGTCGGGGAGAAGAAGATCACCCACCACTGGCACAGGAGTGGCAGCACCATTGGAAAAGCATTTGAAGCCACTGTGAGAGGTGCCAAGAGAATGGCAGTCTTGGGAGACACAGCCTGGGACTTTGGATCAGTTGGAGGCGCTCTCAACTCATTGGGCAAGGGCATCCATCAAATTTTTGGAGCAGCTTTCAAATCATTGTTTGGAGGAATGTCCTGGTTCTCACAAATTCTCATTGGAACGTTGCTGATGTGGTTGGGTCTGAACACAAAGAATGGATCTATTTCCCTTATGTGCTTGGCCTTAGGGGGAGTGTTGATCTTCTTATCTCTAGGAGTTGGGGCGGATCAAGGATGCGCCATCAACTTTGGCAAGAGAGAGCTCAAGTGCGGAGATGGTATCTTCATATTTAGAGACTCTGATGACTGGCTGAACAAGTACTCATACTATCCAGAAGATCCTGTGAAGCTTGCATCAATAGTGAAAGCCTCTTTTGAAGAAGGGAAGTGTGGCCTAAATTCAGTTGACTCCCTTGAGCATGAGATGTGGAGAAGCAGGGCAGATGAGATCAATGCCATTTTTGAGGAAAACGAGGTGGACATTTCTGTTGTCGTGCAGGATCCA

Fragment I pCMV_CH17D/ZIKV construct B

CACCCAACTGATCTTCAGCATCTTCAATATTGGCCATTAGCCATATTATTCATTGGTTATATAGCATAAATCAATATTGGCTATTGGCCATTGCATACGTTGTATCTATATCATAATATGTACATTTATATTGGCTCATGTCCAATATGACCGCCATGTTGGCATTGATTATTGACTAGTTATTAATAGTAATCAATTACGGGGTCATTAGTTCATAGCCCATATATGGAGTTCCGCGTTACATAACTTACGGTAAATGGCCCGCCTGGCTGACCGCCCAACGACCCCCGCCCATTGACGTCAATAATGACGTATGTTCCCATAGTAACGCCAATAGGGACTTTCCATTGACGTCAATGGGTGGAGTATTTACGGTAAACTGCCCACTTGGCAGTACATCAAGTGTATCATATGCCAAGTCCGCCCCCTATTGACGTCAATGACGGTAAATGGCCCGCCTGGCATTATGCCCAGTACATGACCTTACGGGACTTTCCTACTTGGCAGTACATCTACGTATTAGTCATCGCTATTACCATGGTGATGCGGTTTTGGCAGTACACCAATGGGCGTGGATAGCGGTTTGACTCACGGGGATTTCCAAGTCTCCACCCCATTGACGTCAATGGGAGTTTGTTTTGGCACCAAAATCAACGGGACTTTCCAAAATGTCGTAATAACCCCGCCCCGTTGACGCAAATGGGCGGTAGGCGTGTACGGTGGGAGGTCTATATAAGCAGAGCTCGTTTAGTGAACCGAGTAAATCCTGTGTGCTAATTGAGGTGCATTGGTCTGCAAATCGAGTTGCTAGGCAATAAACACATTTGGATTAATTTTAATCGTTCGTTGAGCGATTAGCAGAGAACTGACCAGAACATGTCTGGTCGTAAAGCTCAGGGAAAAACCCTGGGCGTCAATATGGTACGACGAGGAGTTCGCTCCTTGTCAAACAAAATAAAACAAAAAACAAAACAAATTGGAAACAGACCTGGACCTTCAAGAGGTGTTCAAGGATTTATCTTTTTCTTTTTGTTCAACATTTTGACTGGAAAAAAGATCACAGCCCACCTAAAGAGGTTGTGGAAAATGCTGGACCCAAGACAAGGCTTGGCTGTTCTAAGGAAAGTCAAGAGAGTGGTGGCCAGTTTGATGAGAGGATTGTCCTCAAGGAAACGCCGTTCCCATGATGTTCTGACTGTGCAATTCCTAATTTTGGGAATGCTGTTGATGACGGGTGGAGCGGAGGTCACTAGACGTGGGAGTGCATACTATATGTACTTGGACAGAAACGACGCTGGGGAGGCCATATCTTTTCCAACCACATTGGGGATGAATAAGTGTTATATACAGATCATGGATCTTGGACACATGTGTGATGCCACCATGAGCTATGAATGCCCTATGCTGGATGAGGGGGTGGAACCAGATGACGTCGATTGTTGGTGCAACACGACGTCAACTTGGGTTGTGTACGGAACCTGCCATCACAAAAAAGGTGAAGCACGGAGATCTAGAAGAGCTGTGACGCTCCCCTCCCATTCCACTAGGAAGCTGCAAACGCGGTCGCAAACCTGGTTGGAATCAAGAGAATACACAAAGCACTTGATTAGAGTCGAAAATTGGATATTCAGGAACCCTGGCTTCGCGTTAGCAGCAGCTGCCATCGCTTGGCTTTTGGGAAGCTCAACGAGCCAAAAAGTCATATACTTGGTCATGATACTGCTGATTGCCCCGGCATACAGCATCAGGTGCATAGGAGTCAGCAATAGGGACTTTGTGGAAGGTATGTCAGGTGGGACTTGGGTTGATGTTGTCTTGGAACATGGAGGTTGTGTCACCGTAATGGCACAGGACAAACCGACTGTCGACATAGAGCTGGTTACAACAACAGTCAGCAACATGGCGGAGGTAAGATCCTACTGCTATGAGGCATCAATATCGGACATGGCTTCGGACAGCCGCTGCCCAACACAAGGTGAAGCCTACCTTGACAAGCAATCAGACACTCAATATGTCTGCAAAAGAACGTTAGTGGACAGAGGCTGGGGAAATGGATGTGGACTTTTTGGCAAAGGGAGCCTGGTGACATGCGCTAAGTTTGCATGCTCCAAGAAAATGACCGGGAAGAGCATCCAGCCAGAGAATCTGGAGTACCGGATAATGCTGTCAGTTCATGGCTCCCAGCACAGTGGGATGATCGTTAATGACACAGGACATGAAACTGATGAGAATAGAGCGAAGGTTGAGATAACGCCCAATTCACCAAGAGCCGAAGCCACCCTGGGGGGTTTTGGAAGCCTAGGACTTGATTGTGAACCGAGGACAGGCCTTGACTTTTCAGATTTGTATTACTTGACTATGAATAACAAGCACTGGTTGGTTCACAAGGAGTGGTTCCACGACATTCCATTACCTTGGCACGCTGGGGCAGACACCGGAACTCCACACTGGAACAACAAAGAAGCACTGGTAGAGTTCAAGGACGCACATGCCAAAAGGCAAACTGTCGTGGTTCTAGGGAGTCAAGAAGGAGCAGTTCACACGGCCCTTGCTGGAGCTCTGGAGGCTGAGATGGATGGTGCAAAGGGAAGGCTGTCCTCTGGCCACTTGAAATGTCGCCTGAAAATGGATAAACTTAGATTGAAGGGCGTGTCATACTCCTTGTGTACCGCAGCGTTCACATTCACCAAGATCCCGGCTGAAACACTGCACGGGACAGTCACAGTGGAGGTACAGTACGCAGGGACAGATGGACCTTGCAAGGTTCCAGCTCAGATGGCGGTGGACATGCAAACTCTGACCCCAGTTGGGAGGTTGATAACCGCTAACCCCGTAATCACTGAAAGCACTGAGAACTCTAAGATGATGCTGGAACTTGATCCACCATTTGGGGACTCTTACATTGTCATAGGAGTCGGGGAGAAGAAGATCACCCACCACTGGCACAGGAGTGGCAGCACCATTGGAAAAGCATTTGAAGCCACTGTGAGAGGTGCCAAGAGAATGGCAGTCTTGGGAGACACAGCCTGGGACTTTGGATCAGTTGGAGGCGCTCTCAACTCATTGGGCAAGGGCATCCATCAAATTTTTGGAGCAGCTTTCAAATCATTGTTTGGAGGAATGTCCTGGTTCTCACAAATTCTCATTGGAACGTTGCTGATGTGGTTGGGTCTGAACACAAAGAATGGATCTATTTCCCTTATGTGCTTGGCCTTAGGGGGAGTGTTGATCTTCTTATCTCTAGGAGTTGGGGCGGATCAAGGATGCGCCATCAACTTTGGCAAGAGAGAGCTCAAGTGCGGAGATGGTATCTTCATATTTAGAGACTCTGATGACTGGCTGAACAAGTACTCATACTATCCAGAAGATCCTGTGAAGCTTGCATCAATAGTGAAAGCCTCTTTTGAAGAAGGGAAGTGTGGCCTAAATTCAGTTGACTCCCTTGAGCATGAGATGTGGAGAAGCAGGGCAGATGAGATCAATGCCATTTTTGAGGAAAACGAGGTGGACATTTCTGTTGTCGTGCAGGATCCA

Fragment I pCMV_CH17D/ZIKV construct C

CACCCAACTGATCTTCAGCATCTTCAATATTGGCCATTAGCCATATTATTCATTGGTTATATAGCATAAATCAATATTGGCTATTGGCCATTGCATACGTTGTATCTATATCATAATATGTACATTTATATTGGCTCATGTCCAATATGACCGCCATGTTGGCATTGATTATTGACTAGTTATTAATAGTAATCAATTACGGGGTCATTAGTTCATAGCCCATATATGGAGTTCCGCGTTACATAACTTACGGTAAATGGCCCGCCTGGCTGACCGCCCAACGACCCCCGCCCATTGACGTCAATAATGACGTATGTTCCCATAGTAACGCCAATAGGGACTTTCCATTGACGTCAATGGGTGGAGTATTTACGGTAAACTGCCCACTTGGCAGTACATCAAGTGTATCATATGCCAAGTCCGCCCCCTATTGACGTCAATGACGGTAAATGGCCCGCCTGGCATTATGCCCAGTACATGACCTTACGGGACTTTCCTACTTGGCAGTACATCTACGTATTAGTCATCGCTATTACCATGGTGATGCGGTTTTGGCAGTACACCAATGGGCGTGGATAGCGGTTTGACTCACGGGGATTTCCAAGTCTCCACCCCATTGACGTCAATGGGAGTTTGTTTTGGCACCAAAATCAACGGGACTTTCCAAAATGTCGTAATAACCCCGCCCCGTTGACGCAAATGGGCGGTAGGCGTGTACGGTGGGAGGTCTATATAAGCAGAGCTCGTTTAGTGAACCGAGTAAATCCTGTGTGCTAATTGAGGTGCATTGGTCTGCAAATCGAGTTGCTAGGCAATAAACACATTTGGATTAATTTTAATCGTTCGTTGAGCGATTAGCAGAGAACTGACCAGAACATGTCTGGTCGTAAAGCTCAGGGAAAAACCCTGGGCGTCAATATGGTACGACGAGGAGTTCGCTCCTTGTCAAACAAAATAAAACAAAAAACAAAACAAATTGGAAACAGACCTGGACCTTCAAGAGGTGTTCAAGGATTTATCTTTTTCTTTTTGTTCAACATTTTGACTGGAAAAAAGATCACAGCCCACCTAAAGAGGTTGTGGAAAATGCTGGACCCAAGACAAGGCTTGGCTGTTCTAAGGAAAGTCAAGAGAGTGGTGGCCAGTTTGATGAGAGGATTGTCCTCAAGGAAACGCCGTGGCGCAGATACTAGTGTCGGAATTGTTGGCCTCCTGCTGACCACAGCTATGGCAGCGGAGGTCACTAGACGTGGGAGTGCATACTATATGTACTTGGACAGAAACGACGCTGGGGAGGCCATATCTTTTCCAACCACATTGGGGATGAATAAGTGTTATATACAGATCATGGATCTTGGACACATGTGTGATGCCACCATGAGCTATGAATGCCCTATGCTGGATGAGGGGGTGGAACCAGATGACGTCGATTGTTGGTGCAACACGACGTCAACTTGGGTTGTGTACGGAACCTGCCATCACAAAAAAGGTGAAGCACGGAGATCTAGAAGAGCTGTGACGCTCCCCTCCCATTCCACTAGGAAGCTGCAAACGCGGTCGCAAACCTGGTTGGAATCAAGAGAATACACAAAGCACTTGATTAGAGTCGAAAATTGGATATTCAGGAACCCTGGCTTCGCGTTAGCAGCAGCTGCCATCGCTTGGCTTTTGGGAAGCTCAACGAGCCAAAAAGTCATATACTTGGTCATGATACTGCTGATTGCCCCGGCATACAGCATCAGGTGCATAGGAGTCAGCAATAGGGACTTTGTGGAAGGTATGTCAGGTGGGACTTGGGTTGATGTTGTCTTGGAACATGGAGGTTGTGTCACCGTAATGGCACAGGACAAACCGACTGTCGACATAGAGCTGGTTACAACAACAGTCAGCAACATGGCGGAGGTAAGATCCTACTGCTATGAGGCATCAATATCGGACATGGCTTCGGACAGCCGCTGCCCAACACAAGGTGAAGCCTACCTTGACAAGCAATCAGACACTCAATATGTCTGCAAAAGAACGTTAGTGGACAGAGGCTGGGGAAATGGATGTGGACTTTTTGGCAAAGGGAGCCTGGTGACATGCGCTAAGTTTGCATGCTCCAAGAAAATGACCGGGAAGAGCATCCAGCCAGAGAATCTGGAGTACCGGATAATGCTGTCAGTTCATGGCTCCCAGCACAGTGGGATGATCGTTAATGACACAGGACATGAAACTGATGAGAATAGAGCGAAGGTTGAGATAACGCCCAATTCACCAAGAGCCGAAGCCACCCTGGGGGGTTTTGGAAGCCTAGGACTTGATTGTGAACCGAGGACAGGCCTTGACTTTTCAGATTTGTATTACTTGACTATGAATAACAAGCACTGGTTGGTTCACAAGGAGTGGTTCCACGACATTCCATTACCTTGGCACGCTGGGGCAGACACCGGAACTCCACACTGGAACAACAAAGAAGCACTGGTAGAGTTCAAGGACGCACATGCCAAAAGGCAAACTGTCGTGGTTCTAGGGAGTCAAGAAGGAGCAGTTCACACGGCCCTTGCTGGAGCTCTGGAGGCTGAGATGGATGGTGCAAAGGGAAGGCTGTCCTCTGGCCACTTGAAATGTCGCCTGAAAATGGATAAACTTAGATTGAAGGGCGTGTCATACTCCTTGTGTACCGCAGCGTTCACATTCACCAAGATCCCGGCTGAAACACTGCACGGGACAGTCACAGTGGAGGTACAGTACGCAGGGACAGATGGACCTTGCAAGGTTCCAGCTCAGATGGCGGTGGACATGCAAACTCTGACCCCAGTTGGGAGGTTGATAACCGCTAACCCCGTAATCACTGAAAGCACTGAGAACTCTAAGATGATGCTGGAACTTGATCCACCATTTGGGGACTCTTACATTGTCATAGGAGTCGGGGAGAAGAAGATCACCCACCACTGGCACAGGAGTGGCAGCACCATTGGAAAAGCATTTGAAGCCACTGTGAGAGGTGCCAAGAGAATGGCAGTCTTGGGAGACACAGCCTGGGACTTTGGATCAGTTGGAGGCGCTCTCAACTCATTGGGCAAGGGCATCCATCAAATTTTTGGAGCAGCTTTCAAATCATTGTTTGGAGGAATGTCCTGGTTCTCACAAATTCTCATTGGAACGTTGCTGATGTGGTTGGGTCTGAACACAAAGAATGGATCTATTTCCCTTATGTGCTTGGCCTTAGGGGGAGTGTTGATCTTCTTATCTCTAGGAGTTGGGGCGGATCAAGGATGCGCCATCAACTTTGGCAAGAGAGAGCTCAAGTGCGGAGATGGTATCTTCATATTTAGAGACTCTGATGACTGGCTGAACAAGTACTCATACTATCCAGAAGATCCTGTGAAGCTTGCATCAATAGTGAAAGCCTCTTTTGAAGAAGGGAAGTGTGGCCTAAATTCAGTTGACTCCCTTGAGCATGAGATGTGGAGAAGCAGGGCAGATGAGATCAATGCCATTTTTGAGGAAAACGAGGTGGACATTTCTGTTGTCGTGCAGGATCCA

Fragment II 17-D_vaccine strain

TTGAGCATGAGATGTGGAGAAGCAGGGCAGATGAGATCAATGCCATTTTTGAGGAAAACGAGGTGGACATTTCTGTTGTCGTGCAGGATCCAAAGAATGTTTACCAGAGAGGAACTCATCCATTTTCCAGAATTCGGGATGGTCTGCAGTATGGTTGGAAGACTTGGGGTAAGAACCTTGTGTTCTCCCCAGGGAGGAAGAATGGAAGCTTCATCATAGATGGAAAGTCCAGGAAAGAATGCCCGTTTTCAAACCGGGTCTGGAATTCTTTCCAGATAGAGGAGTTTGGGACGGGAGTGTTCACCACACGCGTGTACATGGACGCAGTCTTTGAATACACCATAGACTGCGATGGATCTATCTTGGGTGCAGCGGTGAACGGAAAAAAGAGTGCCCATGGCTCTCCAACATTTTGGATGGGAAGTCATGAAGTAAATGGGACATGGATGATCCACACCTTGGAGGCATTAGATTACAAGGAGTGTGAGTGGCCACTGACACATACGATTGGAACATCAGTTGAAGAGAGTGAAATGTTCATGCCGAGATCAATCGGAGGCCCAGTTAGCTCTCACAATCATATCCCTGGATACAAGGTTCAGACGAACGGACCTTGGATGCAGGTACCACTAGAAGTGAAGAGAGAAGCTTGCCCAGGGACTAGCGTGATCATTGATGGCAACTGTGATGGACGGGGAAAATCAACCAGATCCACCACGGATAGCGGGAAAGTTATTCCTGAATGGTGTTGCCGCTCCTGCACAATGCCGCCTGTGAGCTTCCATGGTAGTGATGGGTGTTGGTATCCCATGGAAATTAGGCCAAGGAAAACGCATGAAAGCCATCTGGTGCGCTCCTGGGTTACAGCTGGAGAAATACATGCTGTCCCTTTTGGTTTGGTGAGCATGATGATAGCAATGGAAGTGGTCCTAAGGAAAAGACAGGGACCAAAGCAAATGTTGGTTGGAGGAGTAGTGCTCTTGGGAGCAATGCTGGTCGGGCAAGTAACTCTCCTTGATTTGCTGAAACTCACAGTGGCTGTGGGATTGCATTTCCATGAGATGAACAATGGAGGAGACGCCATGTATATGGCGTTGATTGCTGCCTTTTCAATCAGACCAGGGCTGCTCATCGGCTTTGGGCTCAGGACCCTATGGAGCCCTCGGGAACGCCTTGTGCTGACCCTAGGAGCAGCCATGGTGGAGATTGCCTTGGGTGGCGTGATGGGCGGCCTGTGGAAGTATCTAAATGCAGTTTCTCTCTGCATCCTGACAATAAATGCTGTTGCTTCTAGGAAAGCATCAAATACCATCTTGCCCCTCATGGCTCTGTTGACACCTGTCACTATGGCTGAGGTGAGACTTGCCGCAATGTTCTTTTGTGCCGTGGTTATCATAGGGGTCCTTCACCAGAACTTCAAGGACACCTCCATGCAGAAGACTATACCTCTGGTGGCCCTCACACTCACATCTTACCTGGGCTTGACACAACCTTTTTTGGGCCTGTGTGCATTTCTGGCAACCCGCATATTTGGGCGAAGGAGTATCCCAGTGAATGAGGCACTCGCAGCAGCTGGTCTAGTGGGAGTGCTGGCAGGACTGGCTTTTCAGGAGATGGAGAACTTCCTTGGTCCGATTGCAGTTGGAGGACTCCTGATGATGCTGGTTAGCGTGGCTGGGAGGGTGGATGGGCTAGAGCTCAAGAAGCTTGGTGAAGTTTCATGGGAAGAGGAGGCGGAGATCAGCGGGAGTTCCGCCCGCTATGATGTGGCACTCAGTGAACAAGGGGAGTTCAAGCTGCTTTCTGAAGAGAAAGTGCCATGGGACCAGGTTGTGATGACCTCGCTGGCCTTGGTTGGGGCTGCCCTCCATCCATTTGCTCTTCTGCTGGTCCTTGCTGGGTGGCTGTTTCATGTCAGGGGAGCTAGGAGAAGTGGGGATGTCTTGTGGGATATTCCCACTCCTAAGATCATCGAGGAATGTGAACATCTGGAGGATGGGATTTATGGCATATTCCAGTCAACCTTCTTGGGGGCCTCCCAGCGAGGAGTGGGAGTGGCACAGGGAGGGGTGTTCCACACAATGTGGCATGTCACAAGAGGAGCTTTCCTTGTCAGGAATGGCAAGAAGTTGATTCCATCTTGGGCTTCAGTAAAGGAAGACCTTGTCGCCTATGGTGGCTCATGGAAGTTGGAAGGCAGATGGGATGGAGAGGAAGAGGTCCAGTTGATCGCGGCTGTTCCAGGAAAGAACGTGGTCAACGTCCAGACAAAACCGAGCTTGTTCAAAGTGAGGAATGGGGGAGAAATCGGGGCTGTCGCTCTTGACTATCCGAGTGGCACTTCAGGATCTCCTATTGTTAACAGGAACGGAGAGGTGATTGGGCTGTACGGCAATGGCATCCTTGTCGGTGACAACTCCTTCGTGTCCGCCATATCCCAGACTGAGGTGAAGGAAGAAGGAAAGGAGGAGCTCCAAGAGATCCCGACAATGCTAAAGAAAGGAATGACAACTGTCCTTGATTTTCATCCTGGAGCTGGGAAGACAAGACGTTTCCTCCCACAGATCTTGGCCGAGTGCGCACGGAGACGCTTGCGCACTCTTGTGTTGGCCCCCACCAGGGTTGTTCTTTCTGAAATGAAGGAGGCTTTTCACGGCCTGGACGTGAAATTCCACACACAGGCTTTTTCCGCTCACGGCAGCGGGAGAGAAGTCATTGATGCCATGTGCCATGCCACCCTAACTTACAGGATGTTGGAACCAACTAGGGTTGTTAACTGGGAAGTGATCATTATGGATGAAGCCCATTTTTTGGATCCAGCTAGCATAGCCGCTAGAGGTTGGGCAGCGCACAGAGCTAGGGCAAATGAAAGTGCAACAATCTTGATGACAGCCACACCGCCTGGGACTAGTGATGAATTTCCACATTCAAATGGTGAAATAGAAGATGTTCAAACGGACATACCCAGTGAGCCCTGGAACACAGGGCATGACTGGATCCTGGCTGACAAAAGGCCCACGGCATGGTTCCTTCCATCCATCAGAGCTGCAAATGTCATGGCTGCCTCTTTGCGTAAGGCTGGAAAGAGTGTGGTGGTCCTGAACAGGAAAACCTTTGAGAGAGAATACCCCACGATAAAGCAGAAGAAACCTGACTTTATATTGGCCACTGACATAGCTGAAATGGGAGCCAACCTTTGCGTGGAGCGAGTGCTGGATTGCAGGACGGCTTTTAAGCCTGTGCTTGTGGATGAAGGGAGGAAGGTGGCAATAAAAGGGCCACTTCGTATCTCCGCATCCTCTGCTGCTCAAAGGAGGGGGCGCATTGGGAGAAATCCCAACAGAGATGGAGACTCATACTACTATTCTGAGCCTACAAGTGAAAATAATGCCCACCACGTCTGCTGGTTGGAGGCCTCAATGCTCTTGGACAACATGGAGGTGAGGGGTGGAATGGTCGCCCCACTCTATGGCGTTGAAGGAACTAAAACACCAGTTTCCCCTGGTGAAATGAGACTGAGGGATGACCAGAGGAAAGTCTTCAGAGAACTAGTGAGGAATTGTGACCTGCCCGTTTGGCTTTCGTGGCAAGTGGCCAAGGCTGGTTTGAAGACGAATGATCGTAAGTGGTGTTTTGAAGGCCCTGAGGAACATGAGATCTTGAATGACAGCGGTGAAACAGTGAAGTGCAGGGCTCCTGGAGGAGCAAAGAAGCCTCTGCGCCCAAGGTGGTGTGATGAAAGGGTGTCATCTGACCAGAGTGCGCTGTCTGAATTTATTAAGTTTGCTGAAGGTAGGAGGGGAGCTGCTGAAGTGCTAGTTGTGCTGAGTGAACTCCCTGATTTCCTGGCTAAAAAAGGTGGAGAGGCAATGGATACCATCAGTGTGTTTCTCCACTCTGAGGAAGGCTCTAGGGCTTACCGCAATGCACTATCAATGATGCCTGAGGCAATGACAATAGTCATGCTGTTTATACTGGCTGGACTACTGACATCGGGAATGGTCATCTTTTTCATGTCTCCCAAAGGCATCAGTAGAATGTCTATGGCGATGGGCACAATGGCCGGCTGTGGATATCTCATGTTCCTTGGAGGCGTCAAACCCACTCACATCTCCTATATCATGCTCATATTCTTTGTCCTGATGGTGGTTGTGATCCCCGAGCCAGGGCAACAAAGGTCCATCCAAGACAACCAAGTGGCATACCTCATTATTGGCATCCTGACGCTGGTTTCAGCGGTGGCAGCCAACGAGCTAGGCATGCTGGAGAAAACCAAAGAGGACCTCTTTGGGAAGAAGAACTTAATTCCATCTAGTGCTTCACCCTGGAGTTGGCCGGATCTTGACCTGAAGCCAGGAGCTGCCTGGACAGTGTACGTTGGCATTGTTACAATGCTCTCTCCAATGTTGCACCACTGGATCAAAGTCGAATATGGCAACCTGTCTCTGTCTGGAATAGCCCAGTCAGCCTCAGTCCTTTCTTTCATGGACAAGGGGATACCATTCATGAAGATGAATATCTCGGTCATAATGCTGCTGGTCAGTGGCTGGAATTCAATAACAGTGATGCCTCTGCTCTGTGGCATAGGGTGCGCCATGCTCCACTGGTCTCTCATTTTACCTGGAATCAAAGCGCAGCAGTCAAAGCTTGCACAGAGAAGGGTGTTCCATGGCGTTGCCAAGAACCCTGTGGTTGATGG

Fragment III 17-D_vaccine strain_HDR/SV40pA

TTACCTGGAATCAAAGCGCAGCAGTCAAAGCTTGCACAGAGAAGGGTGTTCCATGGCGTTGCCAAGAACCCTGTGGTTGATGGGAATCCAACAGTTGACATTGAGGAAGCTCCTGAAATGCCTGCCCTTTATGAGAAGAAACTGGCTCTATATCTCCTTCTTGCTCTCAGCCTAGCTTCTGTTGCCATGTGCAGAACGCCCTTTTCATTGGCTGAAGGCATTGTCCTAGCATCAGCTGCCTTAGGGCCGCTCATAGAGGGAAACACCAGCCTTCTTTGGAATGGACCCATGGCTGTCTCCATGACAGGAGTCATGAGGGGGAATCACTATGCTTTTGTGGGAGTCATGTACAATCTATGGAAGATGAAAACTGGACGCCGGGGGAGCGCGAATGGAAAAACTTTGGGTGAAGTCTGGAAGAGGGAACTGAATCTGTTGGACAAGCGACAGTTTGAGTTGTATAAAAGGACCGACATTGTGGAGGTGGATCGTGATACGGCACGCAGGCATTTGGCCGAAGGGAAGGTGGACACCGGGGTGGCGGTCTCCAGGGGGACCGCAAAGTTAAGGTGGTTCCATGAGCGTGGCTATGTCAAGCTGGAAGGTAGGGTGATTGACCTGGGGTGTGGCCGCGGAGGCTGGTGTTACTACGCTGCTGCGCAAAAGGAAGTGAGTGGGGTCAAAGGATTTACTCTTGGAAGAGACGGCCATGAGAAACCCATGAATGTGCAAAGTCTGGGATGGAACATCATCACCTTCAAGGACAAAACTGATATCCACCGCCTAGAACCAGTGAAATGTGACACCCTTTTGTGTGACATTGGAGAGTCATCATCGTCATCGGTCACAGAGGGGGAAAGGACCGTGAGAGTTCTTGATACTGTAGAAAAATGGCTGGCTTGTGGGGTTGACAACTTCTGTGTGAAGGTGTTAGCTCCATACATGCCAGATGTTCTCGAGAAACTGGAATTGCTCCAAAGGAGGTTTGGCGGAACAGTGATCAGGAACCCTCTCTCCAGGAATTCCACTCATGAAATGTACTACGTGTCTGGAGCCCGCAGCAATGTCACATTTACTGTGAACCAAACATCCCGCCTCCTGATGAGGAGAATGAGGCGTCCAACTGGAAAAGTGACCCTGGAGGCTGACGTCATCCTCCCAATTGGGACACGCAGTGTTGAGACAGACAAGGGACCCCTGGACAAAGAGGCCATAGAAGAAAGGGTTGAGAGGATAAAATCTGAGTACATGACCTCTTGGTTTTATGACAATGACAACCCCTACAGGACCTGGCACTACTGTGGCTCCTATGTCACAAAAACCTCAGGAAGTGCGGCGAGCATGGTAAATGGTGTTATTAAAATTCTGACATATCCATGGGACAGGATAGAGGAGGTCACAAGAATGGCAATGACTGACACAACCCCTTTTGGACAGCAAAGAGTGTTTAAAGAAAAAGTTGACACCAGAGCAAAGGATCCACCAGCGGGAACTAGGAAGATCATGAAAGTTGTCAACAGGTGGCTGTTCCGCCACCTGGCCAGAGAAAAGAACCCCAGACTGTGCACAAAGGAAGAATTTATTGCAAAAGTCCGAAGTCATGCAGCCATTGGAGCTTACCTGGAAGAACAAGAACAGTGGAAGACTGCCAATGAGGCTGTCCAAGACCCAAAGTTCTGGGAACTGGTGGATGAAGAAAGGAAGCTGCACCAACAAGGCAGGTGTCGGACTTGTGTGTACAACATGATGGGGAAAAGAGAGAAGAAGCTGTCAGAGTTTGGGAAAGCAAAGGGAAGCCGTGCCATATGGTATATGTGGCTGGGAGCGCGGTATCTTGAGTTTGAGGCCCTGGGATTCCTGAATGAGGACCATTGGGCTTCCAGGGAAAACTCAGGAGGAGGAGTGGAAGGCATTGGCTTACAATACCTAGGATATGTGATCAGAGACCTGGCTGCAATGGATGGTGGTGGATTCTACGCGGATGACACCGCTGGATGGGACACGCGCATCACAGAGGCAGACCTTGATGATGAACAGGAGATCTTGAACTACATGAGCCCACATCACAAAAAACTGGCACAAGCAGTGATGGAAATGACATACAAGAACAAAGTGGTGAAAGTGTTGAGACCAGCCCCAGGAGGGAAAGCCTACATGGATGTCATAAGTCGACGAGACCAGAGAGGATCCGGGCAGGTAGTGACTTATGCTCTGAACACCATCACCAACTTGAAAGTCCAATTGATCAGAATGGCAGAAGCAGAGATGGTGATACATCACCAACATGTTCAAGATTGTGATGAATCAGTTCTGACCAGGCTGGAGGCATGGCTCACTGAGCACGGATGTAACAGACTGAAGAGGATGGCGGTGAGTGGAGACGACTGTGTGGTCCGGCCCATCGATGACAGGTTCGGCCTGGCCCTGTCCCATCTCAACGCCATGTCCAAGGTTAGAAAGGACATATCTGAATGGCAGCCATCAAAAGGGTGGAATGATTGGGAGAATGTGCCCTTCTGTTCCCACCACTTCCATGAACTACAGCTGAAGGATGGCAGGAGGATTGTGGTGCCTTGCCGAGAACAGGACGAGCTCATTGGGAGAGGAAGGGTGTCTCCAGGAAACGGCTGGATGATCAAGGAAACAGCTTGCCTCAGCAAAGCCTATGCCAACATGTGGTCACTGATGTATTTTCACAAAAGGGACATGAGGCTACTGTCATTGGCTGTTTCCTCAGCTGTTCCCACCTCATGGGTTCCACAAGGACGCACAACATGGTCGATTCATGGGAAAGGGGAGTGGATGACCACGGAAGACATGCTTGAGGTGTGGAACAGAGTATGGATAACCAACAACCCACACATGCAGGACAAGACAATGGTGAAAAAATGGAGAGATGTCCCTTATCTAACCAAGAGACAAGACAAGCTGTGCGGATCACTGATTGGAATGACCAATAGGGCCACCTGGGCCTCCCACATCCATTTGGTCATCCATCGTATCCGAACGCTGATTGGACAGGAGAAATACACTGACTACCTAACAGTCATGGACAGGTATTCTGTGGATGCTGACCTGCAACTGGGTGAGCTTATCTGAAACACCATCTAACAGGAATAACCGGGATACAAACCACGGGTGGAGAACCGGACTCCCCACAACCTGAAACCGGGATATAAACCACGGCTGGAGAACCGGACTCCGCACTTAAAATGAAACAGAAACCGGGATAAAAACTACGGATGGAGAACCGGACTCCACACATTGAGACAGAAGAAGTTGTCAGCCCAGAACCCCACACGAGTTTTGCCACTGCTAAGCTGTGAGGCAGTGCAGGCTGGGACAGCCGACCTCCAGGTTGCGAAAAACCTGGTTTCTGGGACCTCCCACCCCAGAGTAAAAAGAACGGAGCCTCCGCTACCACCCTCCCACGTGGTGGTAGAAAGACGGGGTCTAGAGGTTAGAGGAGACCCTCCAGGGAACAAATAGTGGGACCATATTGACGCCAGGGAAAGACCGGAGTGGTTCTCTGCTTTTCCTCCAGAGGTCTGTGAGCACAGTTTGCTCAAGAATAAGCAGACCTTTGGATGACAAACACAAAACCACTGGCCGGCATGGTCCCAGCCTCCTCGCTGGCGCCGGCTGGGCAACATTCCGAGGGGACCGTCCCCTCGGTAATGGCGAATGGGACTCGCGACAGACATGATAAGATACATTGATGAGTTTGGACAAACCACAACTAGAATGCAGTGAAAAAAATGCTTTATTTGTGAAATTAAGCGCTGGCATTGACCCTGAG
